# Children's Evaluations and Expectations of Forgiveness Following Second‐ and Third‐Party Interventions

**DOI:** 10.1111/cdev.70030

**Published:** 2025-08-21

**Authors:** Abby McLaughlin, Julia Marshall, Isabela Gonzalez‐Rubio Saab, Katherine McAuliffe

**Affiliations:** ^1^ Department of Psychology and Neuroscience Boston College Chestnut Hill Massachusetts USA; ^2^ Department of Cognitive and Psychological Sciences Brown University Providence Rhode Island USA; ^3^ Department of Psychology Harvard University Cambridge Massachusetts USA

**Keywords:** forgiveness, justice, social development

## Abstract

Following a transgression, forgiveness can restore power imbalances and repair damaged bonds, helping maintain important relationships. Yet, we know little about which kinds of responses to transgression best foster forgiveness. Across two studies, with 5‐ to 9‐year‐olds in the United States (*N* = 302; 159 female, 64.2% White, tested in 2022 and 2023), we explore children's evaluations of intervention strategies and their expectations of forgiveness by victims. Our key manipulations were intervention type (compensation, punishment, pardoning, or doing nothing) and intervener role (authority figure, peer, or victim responder; Study 2 only). Our findings show that children's expectations of forgiveness and evaluations depend on *who* intervenes and *how*, shedding new light on the relationship between justice‐oriented interventions and forgiveness in childhood.

Although interpersonal conflicts are often dyadic, responses to conflict typically involve more than just the victim and offender; rather, third parties, particularly authority figures, play a central role in how transgressions are responded to and resolved. This is true across development, meaning that even young children must learn how to integrate information about third‐party interventions into their decisions following an interpersonal transgression (e.g., Bustamante et al. 2021). While the specific goals of third‐party interveners may vary, many interventions are aimed at righting wrongs, helping repair damaged relationships, and restoring interpersonal harmony and thus can be broadly conceived of as justice‐oriented interventions. These interventions may aim to reestablish the power imbalance between victim and offender by returning or granting resources to the victim, punishing or otherwise imposing consequences for the offender, or pardoning (forgiving) the offender.

By at least preschool age, children experience interpersonal conflicts with siblings (e.g., Perlman et al. 2007) and with classmates in school settings (e.g., Chen et al. 2001), after which parents and teachers may intervene (Ross 1996; Skoglund 2019). Accordingly, childhood represents a period during which conflict, forgiveness, and various intervention strategies are coming together for the first time (Bustamante et al. 2021; Oostenbroek and Vaish 2019a). This allows us to address important questions about how interventions by third parties influence children's abilities to repair damaged relationships and engage in reconciliation, a process that is often marked by forgiveness. In this introduction, we review key components of this pathway, beginning with work on forgiveness, the central focus of this study, and then moving on to work on interventions and their relationship with forgiveness.

## 1 Forgiveness

Forgiveness has been defined by influential psychologists as “a suite of prosocial motivational changes [toward the transgressor]” (McCullough 2001) or as “a forswearing of negative affect and judgment, by viewing the wrongdoer with compassion and love” (Enright 2014). Although forgiveness need not imply that victims and transgressors have reconciled, forgiveness often plays an important role in this process and can facilitate improved relationships between conflicted parties (McCullough 2001). Children show a sophisticated understanding of forgiveness: Recent research has found that young children, adolescents, and adults have nuanced conceptualizations of forgiveness and differentiate between the consequences associated with forgiveness, punishment, and inaction following transgressions (McLaughlin et al. 2024). Across the lifespan, individuals expect positive consequences, such as empathy and prosocial behavior toward the offender, to follow forgiveness, and expect negative consequences, such as antisocial behavior and avoidance of the offender, to be less likely. Moreover, this study showed that children ages 5–9 understand the term “forgiveness” in adult‐like ways. Additional work from Toda et al. (2024) found that children as young as 4 also exhibit understanding of the functions of forgiveness (operationalized as the acceptance of an apology) in restoring damaged relationships and promoting positive emotions.

Other research on children's forgiveness has shown that children demonstrate an early‐emerging understanding of forgiveness that is sensitive to contextual factors, such as remorse, apology, and intentionality (Amir et al. 2021; van der Wal et al. 2017; Vera Cruz et al. 2024). Studies suggest that, by age 4, children share more resources with apologetic or remorseful transgressors than transgressors who do not show remorse (Oostenbroek and Vaish 2019a); in fact, they forgive at relatively high rates (Amir et al. 2021), a finding that has been replicated across diverse cultural contexts (Amir et al. 2024). Previous research has also shown that children prefer forgiving victims over nonforgiving victims (Oostenbroek and Vaish 2019b). Collectively, this work speaks to the function that forgiveness plays in children's lives from an early age and points to the value of further research into beliefs about when victims will, or ought to, forgive perpetrators. Children's expectations and evaluations of forgiveness may also be sensitive to information about how third parties and victims themselves respond to interpersonal transgressions. Moreover, various interventions may be perceived more or less fair, which may then, in turn, influence children's evaluations of victim forgiveness.

## 2 Justice‐Oriented Interventions and Forgiveness

The types of justice‐oriented interventions pursued by third‐party authority figures likely have implications for children's post‐transgression attitudes and behavior and may, in particular, play a role in their views on forgiveness. For instance, if one classmate has stolen from another, children may think that forgiveness is more likely if a teacher intervenes to compensate the victim or to punish the thief than if the teacher lets the thief off the hook. Interventions by third parties may be even more critical to children's perceptions of justice and willingness to forgive than adults', both because children often have less power to enact justice in interpersonal conflicts and because children may conceive of forgiveness as more tied to reparations (e.g., Enright et al. 1989).

While the relationship between interventions and forgiveness has not yet been explored in development, a growing body of research with adult participants demonstrates the existence of a relationship between justice‐oriented interventions and forgiveness. These studies have shown that both priming justice and implementing interventions that increase perceptions of justice (e.g., punishment or compensation) lead adults to be more likely to forgive (Strelan 2018; Wenzel and Okimoto 2013; Karremans and Van Lange 2005). These findings provide evidence against some frameworks that have defined forgiveness in terms of the absence of punishment and revenge‐seeking (e.g., McCullough et al. 2013) by showing how pursuing and achieving justice can be effective in promoting forgiveness. Support for the idea that forgiveness serves an important function in repairing damaged social bonds and cooperative relationships has come from work showing that forgiveness leads to positive outcomes for victims, including improved physical and psychological health and increased self‐esteem (Riek and Mania 2012; Griffin et al. 2015; van der Wal et al. 2017). However, forgiveness can also have negative consequences for victims when not preceded by justice‐oriented interventions or amends from transgressors, and nonforgiveness is sometimes beneficial for victims (Jones Ross et al. 2018). For example, work has shown that victims who forgive are sometimes perceived as less competent by bystanders (e.g., DiDonato et al. 2014; Smith et al. 2014) and that forgiveness in the absence of transgressor amends can damage victims' self‐respect and self‐concept clarity (Luchies et al. 2010). Further examination of how interventions can promote victim forgiveness and the restoration of healthy relationships while avoiding negative consequences has important implications for policy, education, and parenting.

In addition to these findings on justice‐oriented interventions and interpersonal forgiveness, some studies in the context of interpersonal and intergroup reconciliation also suggest that particular interventions (such as paying reparations or making apologies) can lead participants to view victims or victim groups as obligated to forgive or reconcile with perpetrators or perpetrator groups (Thai et al. 2023; Zaiser and Giner‐Sorolla 2013). This finding contradicts the view of forgiveness as a supererogatory (i.e., not morally obligatory but still morally good) act, as it is described by many philosophers (Gamlund 2010; Calhoun 1992) as well as the majority of psychologists (Enright 1991). If there is a perceived obligation to forgive, this may mean that victims who choose not to forgive may be judged negatively or even viewed as having transgressed themselves (Thai et al. 2023). This relationship between interventions and evaluations of victims has important implications for how we think about justice and thus deserves more attention in psychological research, particularly from a developmental lens.

The existing research with adult participants provides robust evidence that justice‐oriented interventions can serve to promote victim forgiveness and perhaps also lead victims to be perceived as obligated to forgive. While there is no existing research investigating children's intuitions about how justice‐oriented interventions relate to forgiveness, we have observational and experimental evidence that children have some intuitions related to these interventions more generally. This evidence can shed light on children's broader beliefs about responses to transgressions, which may, in turn, relate to their expectations and evaluations of forgiveness.

Observational and self‐report studies from both parents and teachers provide robust evidence that authority figures do intervene in interpersonal conflicts between children, and often do so by punishing perpetrators, providing comfort or compensation to victims, and by engaging in explicit teaching about norms (e.g., Bustamante et al. 2021; Hoyt et al. 2005; Nucci and Nucci 1982). Moreover, studies show that children actively solicit interventions from authority figures: they engage in tattling to teachers or parents about interpersonal transgressions or norm violations (Ingram and Bering 2010; Yucel and Vaish 2018). These findings suggest that children may have normative beliefs about how third‐party authority figures ought to respond to interpersonal transgressions between children and that children may also take these interventions into account as they navigate subsequent interactions with an offender.

Studies have also demonstrated that children employ and positively evaluate both compensation and punishment as third‐party bystanders to transgressions (Marshall and McAuliffe 2022) and engage in second‐party punishment (e.g., punishing when they themselves are victimized) (Gummerum and Chu 2014; McAuliffe et al. 2017). In third‐party contexts, experimental studies have shown that children prefer helpers—those who compensate unfairness (Lee & Lee and Warneken 2020)—yet opt to punish rather than compensate in their behavioral responses to unfairness (McAuliffe and Dunham 2021). Additional studies have independently documented positive evaluations of both third‐party compensation (Riedl et al. 2015) and punishment (Bregant et al. 2016), though other work shows that children may negatively evaluate second‐party punishment (Strauß and Bondü 2022). Children also have different expectations regarding interventions for different figures. By age 6, children in the US viewed adults as more obligated to punish antisocial actors than they viewed peers, suggesting a shift toward an understanding of punishment (and intervention more broadly) as role‐dependent with age (Marshall et al. 2020). Taken together, existing research provides evidence that children have beliefs about when and how agents, including authority figures, bystanders, and victims themselves, ought to respond to transgressions, which may in turn predict their expectations and evaluations of victim forgiveness following interventions.

## 3 The Current Study

In the current studies, we explore how both *who* is intervening and *how* they are intervening affect children's beliefs about forgiveness. We are specifically interested in how children reason about the likelihood of forgiveness following different justice‐oriented interventions. To fully examine how justice‐oriented interventions relate to children's forgiveness, we need to look at children's *expectations* of when it will occur and beliefs about when it *ought* to occur—an approach that has been deployed in other contexts. This approach has been used to accumulate evidence that children positively evaluate and expect third‐party punishment in response to a variety of transgressions, as described above (for a review, see Marshall and McAuliffe 2022), and also positively evaluate and expect helping of victims of antisocial acts (e.g., Marshall et al. 2020). In the current study, we drew on similar strategies to determine which factors play a role in children's expectations of and evaluations of forgiveness, which may then, in turn, predict children's forgiveness‐related attitudes and behaviors. To do so, we presented participants with vignettes of interpersonal transgressions between two children and assessed their beliefs about the likelihood of forgiveness, the victim's obligation to forgive, and evaluations of nonforgiveness, in addition to the fairness of the intervention and the emotions of victims and offenders (in Study 1).

Gaining insight into these attitudes and behaviors early in development will help us to better understand the starting point for individuals' later views of forgiveness and also allow us to identify critical periods for forgiveness‐related interventions and education. Childhood, and in particular the early elementary years, is an important developmental period during which abilities to respond to injustice, resolve conflicts, and manage relationships are emerging, making it important to understand how children perceive interventions from authority figures and calibrate their own resolution strategies in the aftermath of these interventions (McAuliffe et al. 2025; Ross 1996). Moreover, studying children's obligation and morality judgments allows us to shed light on how victims of transgressions are perceived after various interventions and what the consequences may be for victims who withhold forgiveness. Together, the findings of these studies can expand our understanding of how children perceive the relationship between justice and forgiveness and how interventions may shape both their expectations and evaluations of forgiveness.

## 4 Study 1

In our first preregistered study (link), we sought to understand whether *how* third parties intervene influences children's expectations and evaluations of forgiveness. Specifically, we sought to investigate the relationship between justice‐oriented interventions by third‐party authority figures and children's evaluations of the likelihood of victim forgiveness, obligation to forgive, and evaluations of nonforgiveness. We presented children with four vignettes depicting interpersonal transgressions between two children, witnessed by a teacher, and measured their expectations and evaluations after the teacher compensated the victim, punished the transgressor, pardoned the transgressor, or did nothing. We chose to include pardoning as an intervention because, although this response strategy is not widely studied, it remains an important way for adults to help children resolve conflicts and has been the subject of debates in philosophy (MacLachlan 2017; Murphy and Hampton 1988). We sought to determine whether children may believe that, since authority figures are in charge of enforcing rules, they are also in a position to forgive rule violations, or that forgiveness is in the domain of the victim.

### 4.1 Hypotheses

First, we hypothesized that children's forgiveness ratings (likelihood of forgiveness, forgiveness obligation, and evaluations of nonforgiveness [reverse coded]) would depend on the third‐party intervention described in the story (Hypothesis 1). We predicted that children would rate forgiveness‐related items lowest after the teacher did nothing or pardoned; and sought to test whether punishment or compensation was viewed as most likely to lead to forgiveness.

Second, we hypothesized that children's ratings of victim and offender emotions would depend on the teacher's intervention (Hypothesis 2). We expected that victim emotion ratings would be lowest (i.e., least happy) when the teacher pardoned the transgressor or did nothing and sought to test whether punishment or compensation was viewed as most likely to promote positive victim emotions. Similarly, we expected that transgressor emotion scores would be lowest (i.e., least happy) when the third party punished the transgressor and the highest when the third party pardoned the transgressor.

Third, we hypothesized that children's assessment of how fair the intervention is will depend on the third party's intervention (Hypothesis 3). As above, we expected children to rate punishment and compensation as fairer than forgiveness and doing nothing and sought to determine whether children rated punishment or compensation as the fairest.

### 4.2 Methods

#### 4.2.1 Participants

Our sample consisted of *N* = 135 participants, between the ages of 5 and 9, living in the United States. Our sample had a mean age of 7.59 years (SD_age_ = 1.39) and consisted of 68 boys and 67 girls, of which 31 were Asian/Asian American, 7 Black, 14 Hispanic or Latino, 3 Middle Eastern, 1 Native American, 79 White, 1 other, and 1 prefer not to say. Participants were recruited through the online database at (excluded for anonymity) and tested online via Zoom. An additional 10 participants were tested but excluded because of developmental delays disclosed by parents (*n* = 6), because they were accidentally contacted more than once to complete the study (*n* = 3), or because they were younger than 5 years of age (*n* = 1). Participants who were erroneously contacted to complete the study a second time had data from their second session excluded. We began data collection on July 21, 2022.

#### 4.2.2 Study Design

This study design included one within‐subject factor, Intervention (Compensation, Punishment, Pardoning, Doing Nothing). Each Intervention condition was randomly paired with one of four stories, which are described below. All participants were tested in accordance with our IRB‐approved research protocol at Boston College (Protocol #16.242.01).

#### 4.2.3 Procedure

**FIGURE 1:**
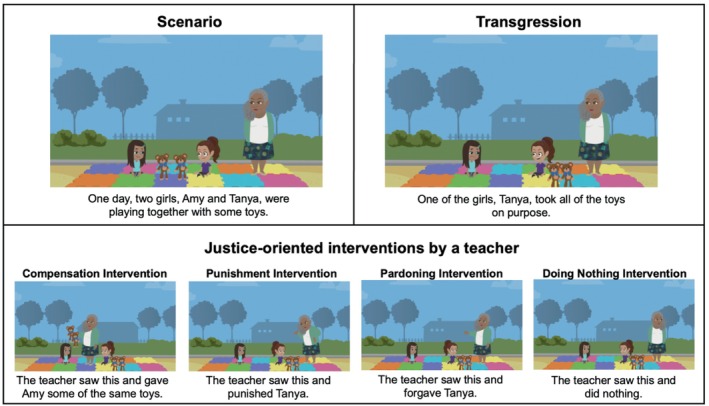
Study 1 Stimuli. Visual depiction of stimuli for one vignette. 2019 GoAnimate Inc. Images are copyrighted by and used by permission of VYOND. VYOND is a trademark of GoAnimate Inc., registered in Australia, Brazil, the European Union, Norway, the Philippines, Singapore, Switzerland, and the United Kingdom.

An experimenter read four illustrated vignettes (Toy Story, Chips Story, Basketball Story, Apple Story), in randomized order, to participants through Zoom (see Figure 1). Each vignette depicted two children, a victim and a transgressor, and a teacher who witnessed the transgression (full materials available in SOM). After being introduced to the characters, participants were told about a theft transgression, wherein the transgressor took an object (toy or food item) belonging to the victim. We chose to focus on theft because these vignettes allowed us to depict compensation as the replacement of lost resources. Next, participants were told that the teacher responded by punishing the transgressor, compensating the victim (by replacing the stolen resource), pardoning (forgiving) the transgressor, or doing nothing. Intervention was randomly paired with story.

Participants first answered a comprehension check question in order to ensure that they understood the intervention; incorrect responses were corrected by the experimenter, and participants were asked again. The vast majority of participants answered correctly either on their first or second attempt (in over 95% of trials). After answering correctly or being reminded of the intervention (after two incorrect answers), participants answered six questions—three about forgiveness (Likelihood of Forgiveness, Evaluations of Nonforgiveness, Obligation to Forgive), two about emotional states (Victim Emotions, Transgressor Emotions) and one about the fairness of the intervention. Questions were separated into three blocks in a fixed order (Forgiveness, Emotions, Fairness) because we were most interested in the Forgiveness questions and did not want exposure to subsequent blocks to alter responses to those questions. The order of questions within each block was randomized.

In the first block (Forgiveness Block), participants were asked three questions assessing their perceptions of the likelihood of victim forgiveness (“Do you think that [victim] will forgive [transgressor]?”), the victim's obligation to forgive (“Do you think that [victim] has to forgive [transgressor]?”), and evaluations of nonforgiveness (“Let's say that [victim] does not forgive [transgressor]. Do you think it is good or bad that [victim] does not forgive [transgressor]?”). This set of questions constituted the forgiveness block; note that children heard the victim and transgressor names for that respective story in all questions. All questions were first presented as a binary (i.e., yes or no, good or bad) and then followed by a question assessing severity or certainty (a tiny bit sure, a little sure, very sure; a tiny bit good, a little good, very good).

In the next block (Emotions Block), participants were asked about the emotional states of the victim (“Do you think [victim] will feel happy, sad, or just okay?”) and the transgressor (“Do you think [transgressor] will feel happy, sad, or just okay?”) following the intervention. Participants were first asked if the transgressor and the victim would feel good or bad as a result of the third‐party intervention and then were asked how good (a tiny bit good, a little good, very good) or how bad (a tiny bit bad, a little bad, or very bad) they would feel.

In the final block (Fairness Block), participants answered one question about the fairness of the intervention. Participants were reminded of the teacher's response and asked if it was a fair thing to do (“The teacher [intervention]. Do you think this was a fair thing to do?”), after which they were asked how sure they were of their answer (a tiny bit sure, a little bit sure, very sure).

After completing these questions for all four stories, participants were asked to rank the four interventions based on which intervention would be most likely to lead to victim forgiveness (see full materials in SOM for more details).

#### 4.2.4 Analyses

Data were logged by live experimenters and recorded in Qualtrics; then later, 27 videos (roughly 20%) were recoded by a second experimenter. In this sample of trials, there was strong agreement between the live and video coding (*κ* = 0.99, *p* < 0.001). All analyses are based on the live coded values. To test our hypotheses, we computed composite rating scores for each forgiveness‐related item, ranging from −2.5 to 2.5, based on binary responses (Yes/No; Good/Bad) and certainty judgments (A tiny bit sure, A little bit sure, Very sure; A tiny bit good/bad, A little good/bad, Very good/bad) for each question. Composite ratings for emotion items (victim emotions and offender emotions) ranged from −3 to +3, based on binary responses (Happy/Sad) and continuous ratings (A tiny bit happy/sad, A little bit happy/sad, Very happy/sad).

In accordance with our preregistration, we first computed Cronbach's alpha for our forgiveness‐related measures (likelihood of forgiveness, evaluations of nonforgiveness, obligation to forgive) and then went on to analyze all items separately since the alpha was below 0.70. For each item, and in accordance with our preregistered data analysis plan, we built logistic mixed effects models with Intervention (Compensate, Punish, Pardon, Do Nothing; within‐subject; reference = Do Nothing) and Age (in months; continuous) as fixed effects and participant ID as a random effect. We first compared our full model, including main effects of predictors of interest, and a null model, containing only the gender and random effects terms, to reduce the chance of type one errors (Forstmeier and Schielzeth 2011). This model comparison was significant for all items (*p* < 0.01).

Our main effects models included the main effects of Intervention and Age. We also built a second model for each item which included the interaction between Age and Intervention for each item and compared our main effects and interaction models to determine whehter the interaction model was a better fit; outputs from these comparisons are included in the SOM (Tables S1–S6).

We also conducted exploratory analyses exploring how fairness ratings predicted ratings for other dependent variables and children's rankings of the fairness of each intervention (Figure S1), which are included in SOM. Data and code are publicly available on OSF.

### 4.3 Results

#### 4.3.1 Likelihood of Forgiveness

**FIGURE 2:**
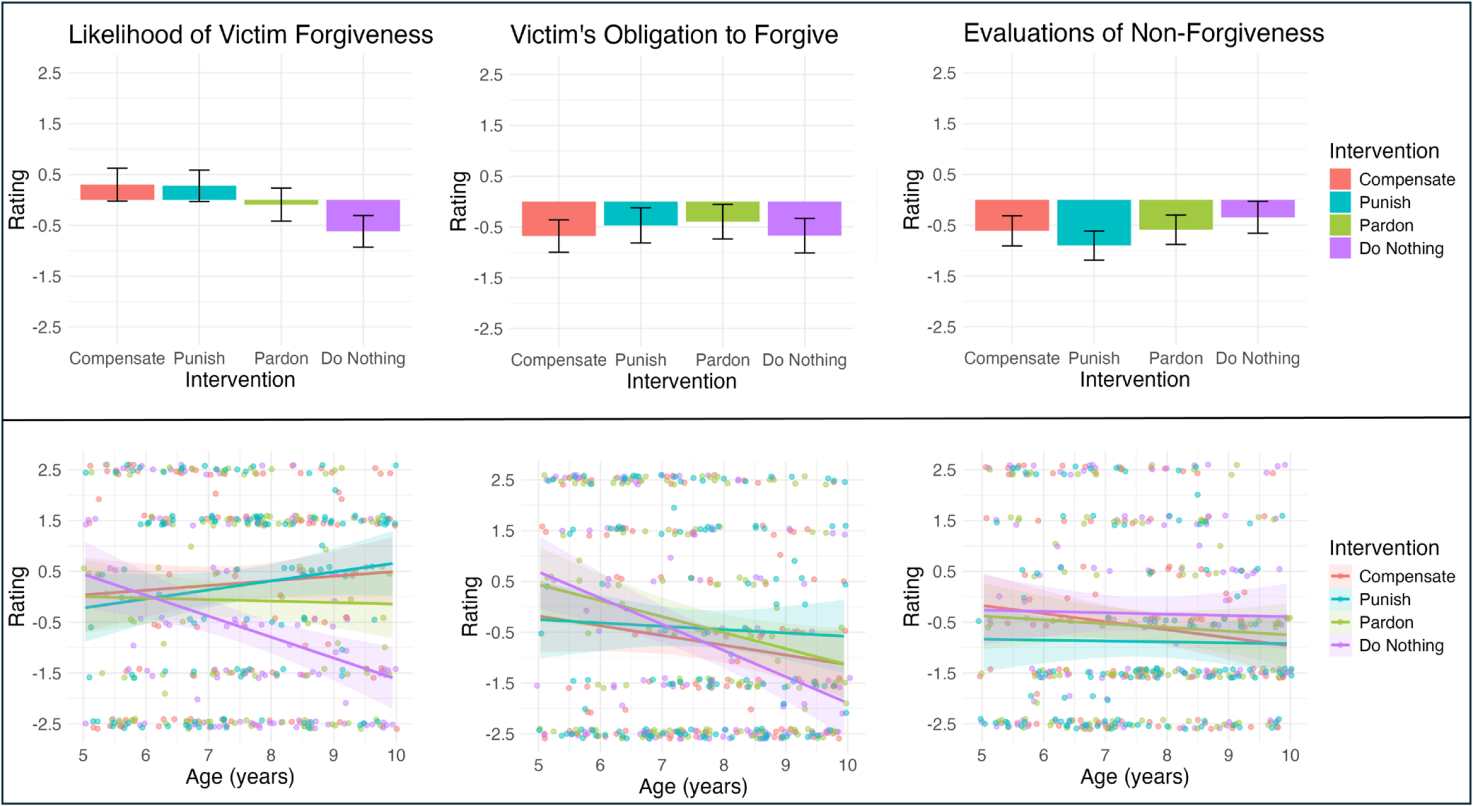
Effect of Intervention and Interaction Effect of Intervention and Age for Likelihood of Forgiveness, Evaluations of Non‐Forgiveness, and Obligation to Forgive. Participant ratings of the Likelihood of Victim Forgiveness, Obligation to Forgive, and Evaluations of Nonforgiveness by Intervention (Compensation, Punishment, Pardoning, Doing Nothing) in Row 1 and by Intervention and Age (continuous) in Row 2. In row 1, the y‐axis shows participant ratings, ranging from‐2.5 (very sure no) to +2.5 (very sure yes) and the x‐axis shows intervention type; in Row 2, the y‐axis shows participant ratings and the x‐axis shows age (in years). Error bars and ribbons represent 95% confidence intervals.

First, for the likelihood of victim forgiveness (Figure 2), participants' ratings differed based on intervention. We examined the model including each of the predictors as main effects. This model revealed that children were more likely to expect victim forgiveness after the teacher compensated (= 0.92, *p* < 0.001), punished (= 0.90, *p* < 0.001), or pardoned (= 0.53, *p* = 0.005) compared to when the teacher did nothing. Thus, both victim‐oriented (i.e., compensation) and transgressor‐oriented (i.e., punishment) interventions increased the perceived likelihood that victims would forgive transgressors.

#### 4.3.2 Evaluations of Nonforgiveness

Participants tended to view nonforgiveness as bad across interventions, although their ratings did differ based on how the teacher intervened (Figure 2). We first examined the model including each of the predictors as main effects. This model showed that children viewed the victim's decision to not forgive as worse after the teacher punished compared to after the teacher did nothing (= −0.56, *p* = 0.001); in contrast, there was not a significant difference between compensation and doing nothing (*𝛽* = −0.27, *p* = 0.069) or between pardoning and doing nothing (*𝛽* = −0.24, *p* = 0.095). This suggests that offender‐focused interventions in particular make the victim's choice not to forgive bad in comparison to no intervention.

#### 4.3.3 Obligation to Forgive

We found that children rated victims as not obligated to forgive transgressors, regardless of how the teacher intervened (Figure 2). We examined the model including each of the predictors as main effects. The output from this model indicated that children did not rate the obligation to forgive differently after the teacher compensated (= −0.01, *p* = 0.968), punished (= 0.20, *p* = 0.275), or pardoned (= 0.27, *p* = 0.135) compared to after the teacher did nothing, suggesting that children do not perceive any of these justice‐oriented interventions as shifting obligations to forgive to the victim. However, we found a main effect of age: as children got older, they tended to report that victims were less obligated to forgive (= −0.02, *p* = 0.002).

#### 4.3.4 Victim and Offender Emotions

**FIGURE 3:**
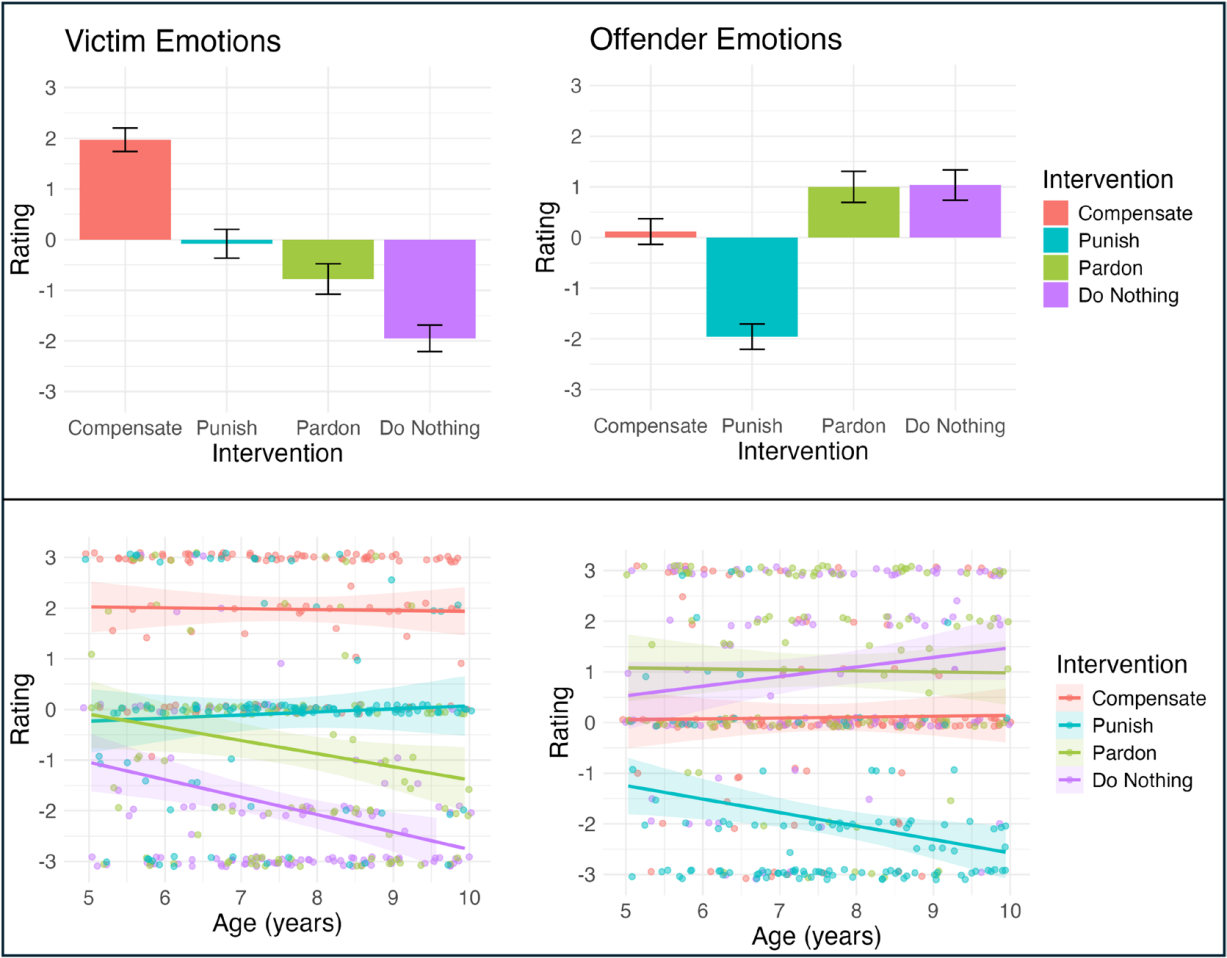
Effect of Intervention and Interaction Effect of Intervention and Age for Victim and Offender Emotions. Participant ratings of Victim Emotions and Offender Emotions by Intervention (Compensate, Punish, Pardon, Do Nothing) in Row 1 and by Intervention and Age (continuous) in Row 2. In Row 1, the y‐axis shows participant ratings, ranging from −3 (very bad) to +3 (very good) and the x‐axis shows intervention type; in Row 2, the y‐axis shows participant ratings, and the x‐axis shows age (in years). Error bars and ribbons represent 95% confidence intervals.

For victim emotions (Figure 3) our participants expected victims to feel different emotions based on how the teacher intervened. We first looked at the model including only main effects, which showed that participants rated victims as happier after the teacher compensated (= 3.92, *p* < 0.001), punished (= 1.87, *p* < 0.001), or pardoned (= 1.17, *p* < 0.001) compared to after the teacher did nothing. In our subsequent pairwise comparisons, we found that children also differentiated between these three interventions in terms of victim emotions; victims were expected to feel happiest after the teacher compensated, followed by after the teacher punished, and then after the teacher pardoned (*p* < 0.001).

**FIGURE 4:**
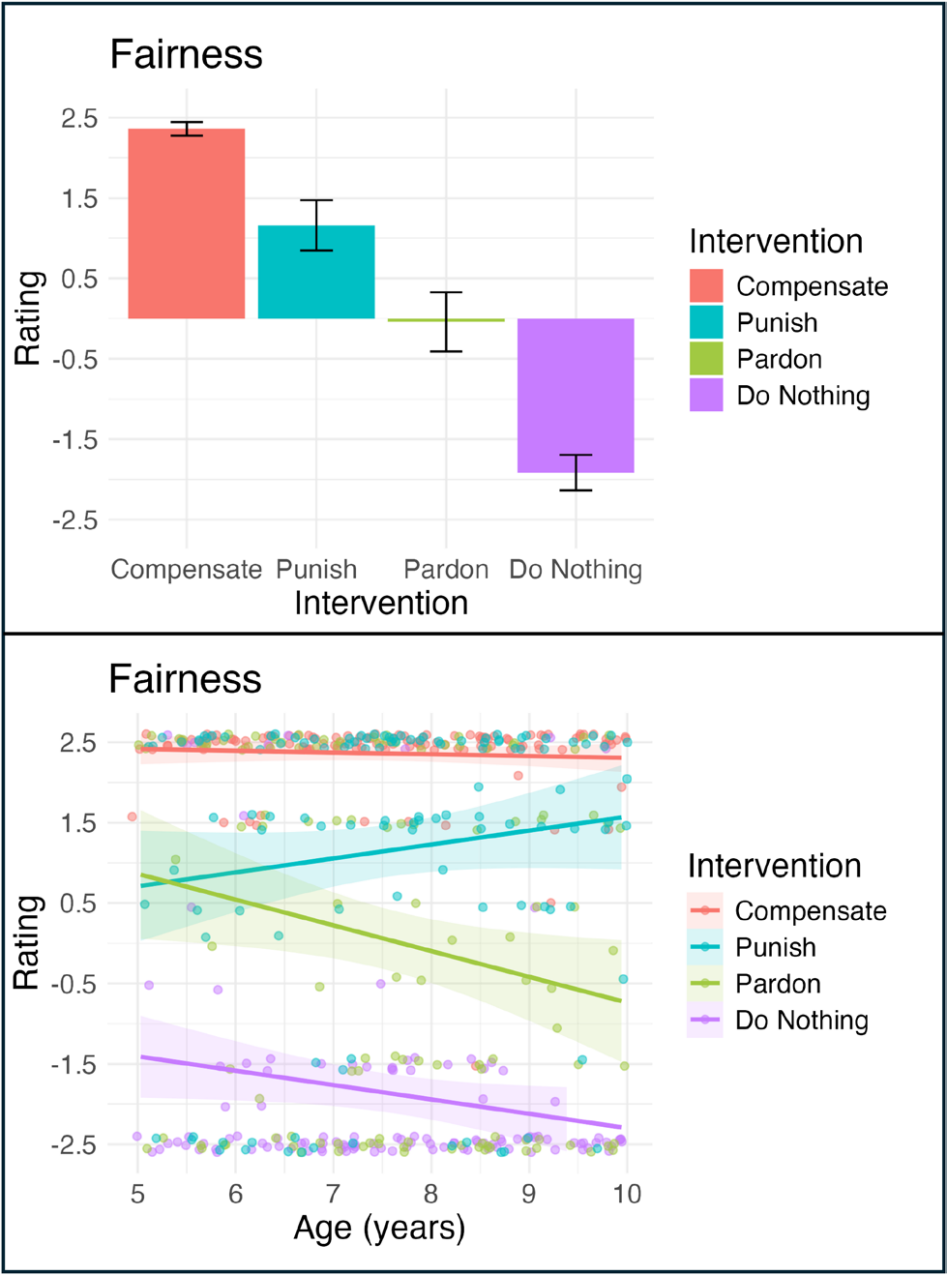
Effect of Intervention and Interaction Effect of Intervention and Age for Fairness. Participant ratings of Fairness by Intervention (Compensation, Punishment, Pardoning, Doing Nothing) on the left and by Intervention and Age (continuous) on the right. On the left, the y‐axis shows participant ratings, ranging from‐2.5 (very sure no) to +2.5 (very sure yes) and the x‐axis shows intervention type; on the right, the y‐axis shows participant ratings and the x‐axis shows age (in years). Error bars represent 95% confidence intervals.

For offender emotions (Figure 4), our participants expected offenders to feel different emotions based on how the teacher intervened. We first examined the model including only main effects, which revealed that participants rated offenders as sadder after the teacher compensated (= −0.92, *p* < 0.001) or punished (= −2.99, *p* < 0.001) compared to after the teacher did nothing. Our subsequent pairwise comparisons showed that children also expected the offender to feel sadder after the teacher punished compared to after they compensated (= 2.07, *p* < 0.001).

#### 4.3.5 Fairness

Finally, for fairness (Figure 4), participants rated the fairness of the four third‐party interventions differently; in particular, our first model, including the main effects of each predictor, showed that children view compensation (= 4.27, *p* < 0.001), punishment (= 3.07, *p* < 0.001), and pardoning (= 1.87, *p* < 0.001) as more fair than doing nothing. Pairwise comparisons revealed that children perceived compensation to be the fairest intervention, followed by punishment, then pardoning, and finally doing nothing (*p* < 0.001).

### 4.4 Discussion

Study 1 shows that children between the ages of 5 and 9 calibrate their evaluations and expectations of victim and transgressor responses to transgressions based on justice‐oriented interventions by third parties, providing partial support of our four hypotheses.

Addressing Hypothesis 1, we found that children calibrate some judgments regarding victim forgiveness based on *how* third parties engaged in justice‐oriented interventions, partially confirming our hypothesis. In particular, children were more likely to expect victims to forgive after the teacher compensated the victim or punished the transgressor compared to after the teacher pardoned the transgressor or did nothing. Children also tended to report that not forgiving was worse after the teacher punished compared to after they did nothing, suggesting that children view the decision not to forgive as particularly bad when the intervention is offender‐focused.

Although the likelihood of forgiveness depended on the intervention, children did not rate the victim's obligation to forgive differently based on how the teacher intervened. Children tended to report that victims were not obligated to forgive, suggesting that forgiveness is not viewed as obligatory in childhood and that third‐party interventions do not meaningfully shift victims' obligations. At the same time, children tended to rate nonforgiveness as bad across all conditions, which seems to be at odds with their obligation judgments. These findings introduce a potential tension in children's views of forgiveness and motivated further investigation in Study 2.

Addressing Hypothesis 2, we found that children's ratings of victim and offender emotions varied in accordance with the teacher's intervention. As we hypothesized, children expected victims to feel happiest after the teacher compensated or punished and expected offenders to be happiest after the teacher pardoned or did nothing. This pattern of results suggests that children expect victims and offenders to respond differently to third‐party interventions and, in particular, expect victims to respond more positively to restorative or punitive interventions and offenders to respond more positively to the maintenance of the status quo.

Addressing Hypothesis 3, we confirmed our prediction that children would view compensation and punishment as fairer than pardoning or doing nothing, and also found that, in the context of third‐party responses to theft violations, victim compensation is viewed as most fair. Our participants tended to rate victim compensation as the fairest intervention, followed by transgressor punishment, and then pardoning and doing nothing. These responses align with previous research showing that children prefer helpers over punishers and also prefer the act of helping over punishing (Lee & Lee and Warneken 2020), and, despite not providing direct evidence, may be interpreted as support for the idea that children hold these views because they perceive compensation as fairer (or more *just*) than other third‐party interventions.

While Study 1 clearly showed that children's expectations of victim forgiveness depend on how third‐party authority figures intervene, we are still left with several outstanding questions, which motivated Study 2. First, it was unclear why children rated nonforgiveness as bad while simultaneously reporting that victims were not obligated to forgive; thus, in Study 2, we sought to replicate these results and determine if contextual factors could alter either of these ratings. Second, we only measured children's evaluations and expectations of forgiveness‐related outcomes in the aftermath of interventions by a teacher, which left us wondering if our pattern of results would hold if other agents responded to a transgression. In Study 2, we included teachers, peers, and victims themselves as agents who could respond to transgressions. By including victims as second‐party interveners, we hoped to build on previous work showing that children evaluate second‐party punishment differently than third‐party punishment (Strauß and Bondü 2022) and test if victim agency in interventions would lead participants to view forgiveness as more obligatory. Third, in Study 1, we only presented children with one transgression type (theft); thus, we sought to understand if children's judgments would differ if asked about transgressions that differed in severity, such as fairness violations. Fourth, in this first study, we told children how teachers responded to transgressions rather than soliciting judgments of how an actor *should* intervene. In Study 2, we assessed children's views of how different actors should respond to transgressions.

## 5 Study 2

In our second preregistered study (link), we examined children's perceptions of the relationship between interventions and forgiveness by manipulating the agent intervening, the type of intervention, and the type of transgression (theft or fairness condition). We presented children with three stories in which a victim, a peer, or a teacher could respond to an interpersonal transgression between two children and first assessed their normative beliefs by asking which intervention they thought the actor should engage in. We then measured evaluations of the fairness of the intervention, the likelihood of victim forgiveness, the victim's obligation to forgive, and evaluations of nonforgiveness. In Study 2, we chose to exclude pardoning as an intervention, as it only made sense for authority figures to pardon, and to exclude the two emotion items, since our hypotheses regarding victim and offender (Hypothesis 2) emotions were confirmed in Study 1.

### 5.1 Hypotheses

Prior to conducting Study 2, we preregistered several hypotheses. First, we hypothesized that children's forgiveness scores (likelihood of forgiveness, forgiveness obligation, and evaluations of how bad it would be not to forgive [reverse coded]) would vary based on the intervention (Hypothesis 1). In particular, we hypothesized that forgiveness scores would be the lowest when the intervener did nothing and then looked to determine whether forgiveness scores would be higher after punishment or compensation. We also hypothesized that the intervention would affect children's assessments of fairness (Hypothesis 2); we hypothesized that children would rate punishment and compensation as fairer than doing nothing.

Furthermore, we predicted that children's forgiveness scores and assessments of fairness would depend on the type of transgression: theft or unfairness (Hypothesis 3). We hypothesized that theft would be viewed as more severe than unfairness; therefore, forgiveness ratings would be higher for unfairness, and punishment would be rated as fairer for theft.

We also hypothesized that children's evaluations of the normative response to transgressions would be influenced by the intervener (Hypothesis 4). For victims and peers, we expected that compensation would be evaluated as the most appropriate response. For teachers, we expected that punishment and compensation would be rated higher than doing nothing; however, we did not have a precise prediction regarding the relationship between punishment and compensation when a teacher acts as an intervener.

### 5.2 Methods

#### 5.2.1 Participants

Our sample consisted of *N* = 167 participants between the ages of 6 and 9 and living in the United States. We chose to limit our sample to 6–9‐year‐olds in Study 2 because some 5‐year‐olds in Study 1 had difficulty sustaining attention throughout the entire task. Our sample had a mean age of 8.04 years (SD_age_ = 1.15) and consisted of 74 boys, 92 girls, and 1 nonbinary participant, of which 38 were Asian or Asian American, 11 Black or African American, 19 Hispanic or Latino, 1 Native American, 2 Pacific Islander, 115 White, and 4 Other/prefer not to say. Participants were recruited through our online database at [school name] and tested online via Zoom. An additional 8 participants were tested but excluded because of developmental delays disclosed by parents (*n* = 1), because they had previously participated in Study 1 (*n* = 4), because they were younger than 6 years of age (*n* = 1), or because they did not finish the study (*n* = 2). We began data collection on December 16, 2023.

#### 5.2.2 Study Design

This study design included two within‐subject factors—Intervention (Punish, Compensate, Do Nothing) and Actor (Teacher, Peer, Victim)—and one between‐subject factor—Transgression Type (Theft, Unfairness). Each Actor condition was randomly paired with one of three stories, which are described below, and children answered questions about all three interventions for each of the three stories. All participants were tested in accordance with our IRB approved research protocol at [excluded for anonymity; Protocol no.: XXXXXX].

#### 5.2.3 Procedure

**FIGURE 5:**
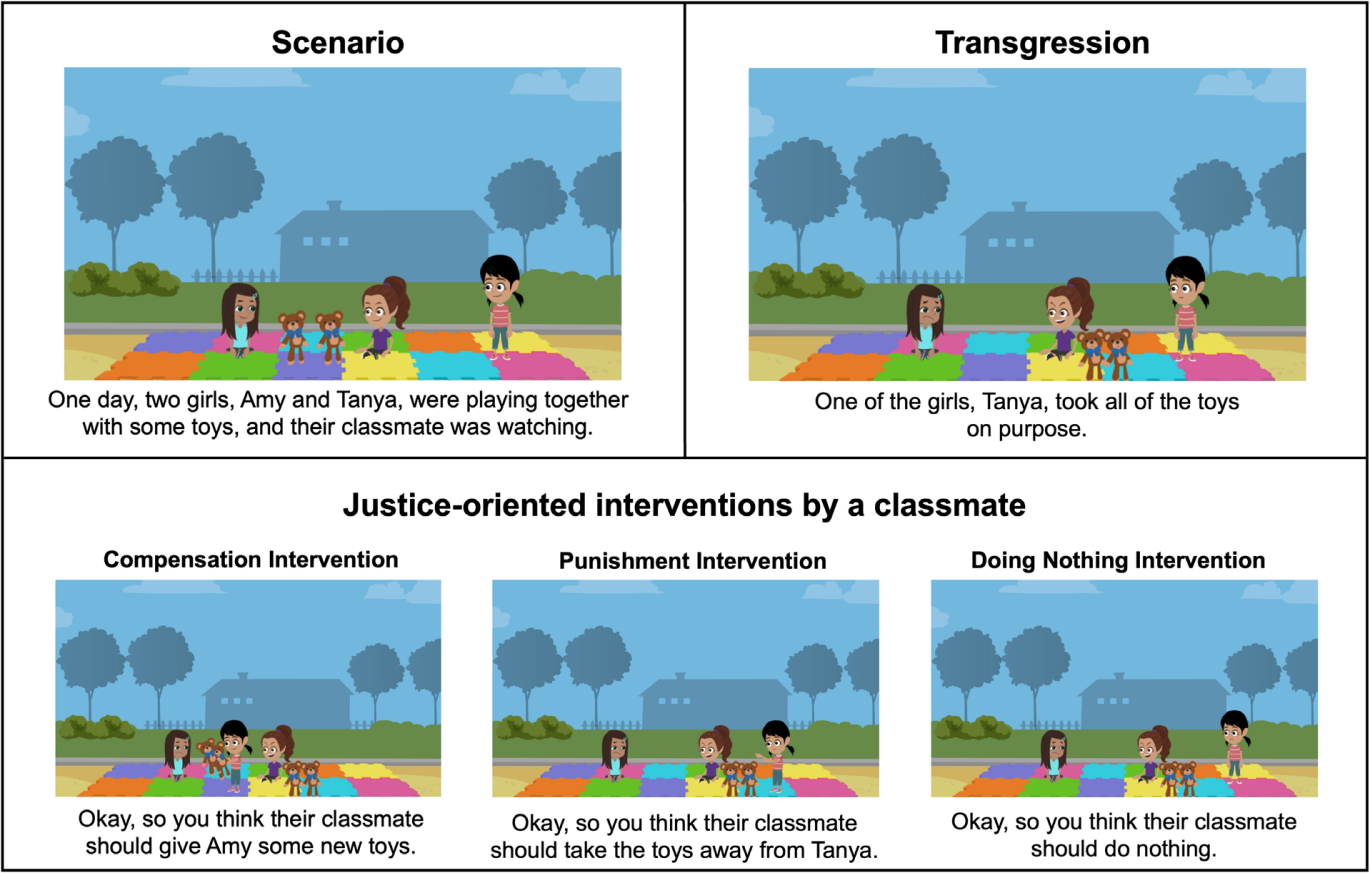
Study 2 Stimuli. Visual depiction of stimuli for one vignette (Peer Condition). Row 1 shows the introduction of the story where the transgression (Toy Theft) was witnessed by a classmate. Row 2 shows intervention choice, where participants were asked if their classmate should punish, compensate, or do nothing in response to the transgression.

An experimenter read three illustrated vignettes (Toy Story, Snack Story, Apple Story), in randomized order, via Zoom. Participants were assigned to either the Theft condition or the Unfairness condition, which meant that they saw vignettes depicting a transgressor taking resources from a victim or a transgressor refusing to share resources with a victim, respectively. In each vignette, participants were introduced to two main characters and were then told about an interpersonal transgression committed by one of the characters (Figure 5). The transgression was witnessed by a teacher (Teacher Condition), a classmate (Peer Condition), or just the two main characters (Victim Condition); Condition was randomly paired with Vignette. After learning about the transgression, participants were asked how the teacher, classmate, or victim should respond to the transgression.

After selecting one of the three interventions, participants answered the first block of questions evaluating the outcomes associated with their selected intervention. For each dependent measure, children were reminded of the intervention before hearing the question. In randomized order, participants were asked about the likelihood of forgiveness, obligation to forgive, evaluations of nonforgiveness, and fairness, using the same items as Study 1.

After participants answered these four questions for the intervention they selected, they were then asked the same questions for the other two interventions. For example, participants were told, “Now, let's imagine that [the teacher/their classmate/the victim] actually chose to [punish/compensate/do nothing]. Now I'll ask you some questions about what you think will happen after [the teacher/their classmate/the victim] [punished/compensated/did nothing].” Participants answered three blocks of these four questions for each of three vignettes. After completing the main task, participants answered a comprehension check question, which asked them if the transgressor in a story took resources or chose not to share resources, and a severity question, which asked them how good or bad taking resources and choosing not to share resources was in the context of one of the vignettes (using a modified 6‐point Likert scale from very bad to very good), as well as which transgression type (Theft versus Unfairness) is worse. The majority (97%) of participants answered comprehension checks correctly in their first two attempts.

#### 5.2.4 Coding and Analysis

Data were logged by live experimenters and recorded in Qualtrics, and then later 27 videos (roughly 20%) were recoded by a second experimenter. There was strong agreement between the live and video coding (*κ* = 0.99, *p* < 0.001). All analyses are based on live coded values.

First, to explore children's normative beliefs about how actors should intervene in response to the transgression, we conducted chi‐squared tests for children's overall choice of intervention and for children's choice of intervention by actor.

Next, to test our hypotheses regarding children's ratings for forgiveness‐related items and fairness, we computed composite rating scores for each item, ranging from‐2.5 to 2.5, based on participants' binary responses (Yes/No; Good/Bad) and certainty judgments (A tiny bit sure, A little bit sure, Very sure; A tiny bit good/bad, A little good/bad, Very good/bad).

In accordance with our preregistration, we first computed Cronbach's alpha for our forgiveness‐related measures (likelihood of forgiveness, evaluations of nonforgiveness, obligation to forgive) and then went on to analyze all items separately since the alpha was below 0.70 (*a* = 0.63). For each item, and in accordance with our preregistered data analysis plan, we built logistic mixed effects models with Intervention (Compensate, Punish, Pardon, Do Nothing; within‐subject; reference = Do Nothing), Actor (Peer, Victim, Teacher; reference = Victim), Transgression Type (Unfairness, Theft; reference = Unfairness), and Age (continuous) as fixed effects. In each model, Gender was included as a fixed effect to control for its influence, and Participant ID was added as a random effect to account for individual variability across participants. Model 1 included the three‐way interaction between Intervention, Actor, and Transgression Type. Model 2 included all two‐way interactions between these variables; Model 3 included only their main effects, and Model 4 (the null model) included only control variables (Gender and Participant ID). Below, we report which model best fits the data; model comparisons were conducted by likelihood ratio tests. Although we do not report four‐way interactions including Age, due to lack of power to detect four‐way interactions, we include these models in the RMarkdown file on OSF. The full model outputs and model comparisons can be found in the SOM (Tables S7–S10). Data and code are publicly available on OSF.

### 5.3 Results

#### 5.3.1 Normative Beliefs About Interventions

**FIGURE 6:**
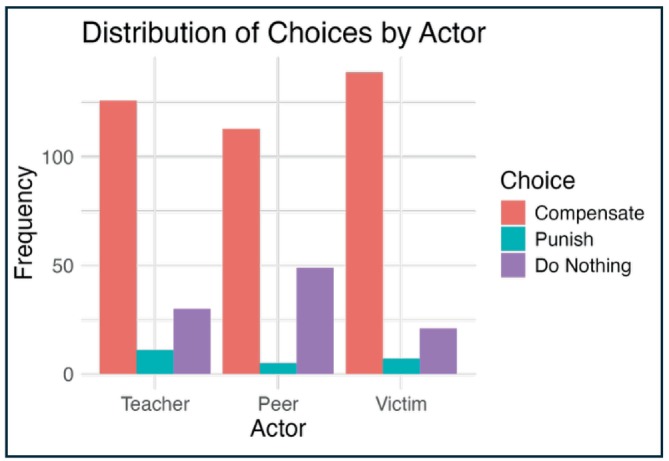
Intervention choices by Actor. Participant selections for the normative intervention (Compensation, Punishment, Doing Nothing) by Actor (Teacher, Peer, Victim). Y‐axis represents the frequency of selection and X‐axis represents the Actor.

To test if children tended to favor some interventions over others in their normative judgments, we conducted chi‐squared tests for children's overall choice of intervention and for children's choice of intervention by actor (Figure 6). We found significant results for both overall choice (*X* ^2^(2) = 417.64, *p* < 0.001) and for choice by actor (*X* ^2^(4) = 17.38, *p* = 0.002), such that children were most likely to select compensation, followed by punishment, and then doing nothing, with participants distinguishing between interventions most when the actor was the victim.

#### 5.3.2 Forgiveness Measures

##### 5.3.2.1 Likelihood of Forgiveness

**FIGURE 7:**
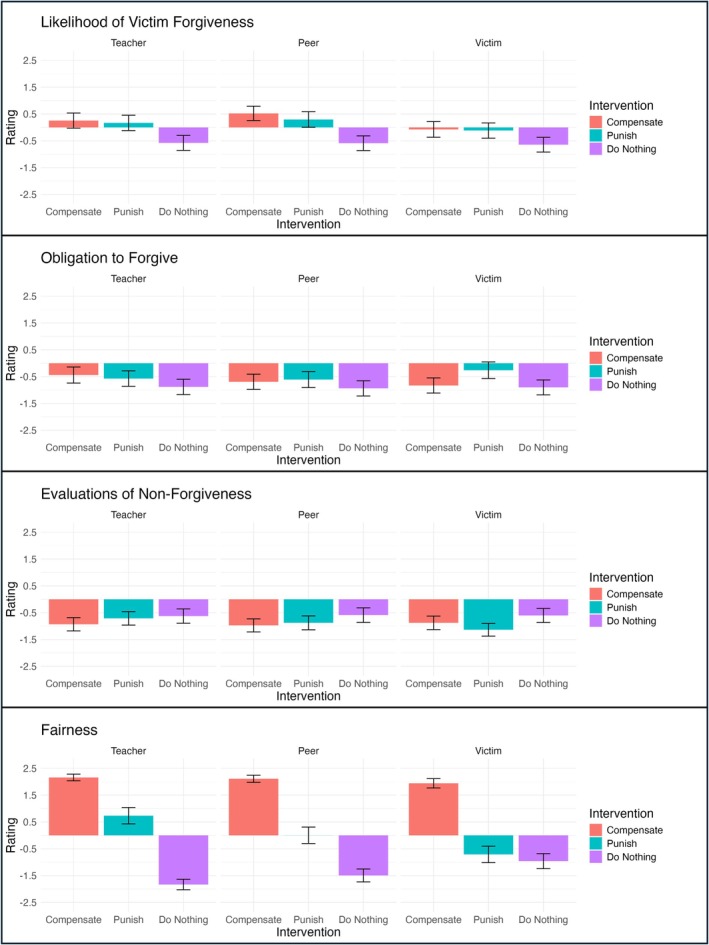
Ratings for Likelihood of Victim Forgiveness, Obligation to Forgive, Evaluations of Non‐Forgiveness, and Fairness by Actor and Intervention. Participant ratings of the likelihood of forgiveness, obligation to forgive, and evaluations of nonforgiveness by Intervention (Compensation, Punishment, Doing Nothing) and Actor (Teacher, Peer, Victim). The y‐axis shows participant ratings, ranging from‐2.5 (very sure no/very bad) to +2.5 (very sure yes/very good) and the x‐axis shows intervention type. Error bars represent 95% confidence intervals.

For the likelihood of victim forgiveness (Figure 7, row 1), Model 3, the main effects model, best fit our data (*X* ^2^ = 104.61, *p* < 0.001). The model showed that children's ratings of the likelihood of victim forgiveness depended on how the actor intervened. In particular, children rated victims as more likely to forgive when an actor compensated (= 0.84, *p* < 0.001) or punished (= 0.72, *p* < 0.001) compared to when the actor did nothing. This finding suggests that children expect victim‐oriented interventions to be most effective in promoting forgiveness in the aftermath of interpersonal transgressions. The model also showed that children's ratings depended on who intervened; children reported that victims were more likely to forgive when the actor was a teacher (= 0.36, *p* < 0.001) or a peer (= 0.22, *p* = 0.019) compared with the victim themselves. We did not find a significant main effect of Transgression Type, suggesting that children's expectations of victim forgiveness did not depend on whether the transgression was theft or unfairness.

##### 5.3.2.2 Evaluations of Nonforgiveness

For evaluations of nonforgiveness (Figure 7, row 2), Model 3, the main effect model, was again the best fit for our data, *X* ^2^(6) = 31.55, *p* < 0.001. This model revealed two main findings: first, children perceived the victim's choice to not forgive was worse after the intervener compensated (= −0.32, *p* < 0.001) or punished (= −0.30, *p* < 0.001). This finding suggests that children think it is worse to refrain from forgiveness after any intervention has occurred, regardless of whether it is a victim‐ or offender‐focused intervention and regardless of who is intervening. Second, children's evaluations of nonforgiveness changed with age; as children got older, they were less likely to report that not forgiving was bad (= 0.15, *p* = 0.027). The main effect of age suggests that children may be developing increasingly nuanced perceptions of forgiveness, such that they increasingly recognize that withholding forgiveness may yield benefits. Again, we did not find a main effect of Transgression Type.

##### 5.3.2.3 Obligation to Forgive

For the victim's obligation to forgive (Figure 7, row 3), we found a somewhat different result than the other items. For this item, Model 2, which included the two‐way interactions between our predictors, was the best model fit, *X* ^2^(8) = 18.35, *p* = 0.019. Model 2 revealed a significant interaction between intervention type and actor; the reference variable for Model 2 was changed to compensation to allow us to identify where effects were emerging. Children differentiated between the victim's obligation to forgive after punishment versus compensation more so when the actor was the teacher (= −0.70, *p* < 0.001) or peer (= −0.49, *p* = 0.016) versus the victim. Victims were rated as more obligated to forgive after the teacher or peer compensated compared to after the teacher or peer punished, whereas ratings were similar when the victim compensated or punished. This finding suggests that children's beliefs about when victims are obligated to forgive depend on both *how* an actor intervenes and *who* is intervening. The model output also showed a main effect of Transgression Type (*𝛽* = 0.75, *p* = 0.002), with participants rating victims as more obligated to forgive when the transgression was theft versus unfairness.

##### 5.3.2.4 Fairness

For fairness (Figure 7), Model 2, which included the two‐way interactions between our predictors, was again the best fit for our data, *X* ^2^(8) = 102.76, *p* < 0.001. This model revealed two key findings. First, we found that children's judgments of the fairness of punishment compared to doing nothing varied based on whether the transgression was theft or unfairness (= 0.46, *p* = 0.016). This interaction effect indicates that children may view offender‐focused interventions, such as punishment, as fairer than inaction when transgressions are more severe (i.e., in the case of theft vs. unfairness). Second, we found that children differentiated between the fairness of punishment and doing nothing more when the actor was a peer (= 1.25, *p* < 0.001) or teacher (= 2.32, *p* < 0.001) compared with the victim, and between the fairness of compensation and doing nothing more when the actor was a peer (= 0.70, *p* = 0.003) or teacher (= 1.09, *p* < 0.001) compared with the victim. This suggests that the intervener role has a major influence on how the fairness of an intervention is evaluated. We did not find a main effect of Transgression Type.

We also analyzed severity ratings to ensure that participants differentiated between unfairness and theft; in fact, we found that children reliably rated theft as worse than unfairness. Full results from these analyses are described in the SOM.

### 5.4 Discussion

Study 2 shows that children between the ages of 6 and 9 differentially evaluate interventions from third‐party authority figures, third‐party peers, and second parties (victims) and form different beliefs about what will and ought to follow these interventions.

Addressing Hypothesis 1, we found that both the intervention and actor had significant effects on children's ratings of forgiveness‐related measures, which included the likelihood of forgiveness, the victim's obligation to forgive, and evaluations of nonforgiveness. In particular, children expected forgiveness to be more likely when the actor compensated or punished compared to when the actor did nothing, as well as when a peer responded compared to when a victim responded. The tendency to view forgiveness as more likely following third‐party compared to second‐party interventions may suggest that children view third parties as more objective arbiters of justice, perceive third‐party interventions as more effective in eliciting changes in the transgressor, or consider victims' interventions to be antithetical to forgiveness. Though the present study cannot determine precisely why we observed this pattern of results, future research might test between these possibilities by eliciting justifications. Children also viewed nonforgiveness as worse after the actor compensated or punished compared to after they did nothing and judged the victim as more obligated to forgive when the actor punished compared to when the actor did nothing, an effect that was more pronounced when that actor was a teacher or peer versus the victim. These findings deviate from the findings of Study 1, but align with our hypotheses, and suggest that both of these judgments are sensitive to information about justice‐related pursuits.

Moreover, replicating the same pattern as Study 1, we found that children viewed nonforgiveness as bad, despite also reporting, on average, that victims were not obligated to forgive. Although these results seem to be at odds with one another, on closer examination, it may be that children are viewing forgiveness as supererogatory, a possibility that we will further unpack in the General Discussion. The results of Study 2 suggest that, even when the transgression is less severe or when the victim themselves has agency in responding to the transgression, forgiveness is not obligatory; although nonforgiveness is still bad.

Addressing Hypothesis 2, by asking children to rate how fair various interventions were when employed by different actors, we were able to gain insight into how our participants perceived each of these actions. As in Study 1, we found that participants viewed compensation as the fairest intervention, followed by punishment, and then doing nothing. However, Study 2 showed that fairness judgments were predicted by an interaction between intervention and actor; in particular, participants viewed punishment as fair from teachers and as unfair from victims. This pattern of results suggests that children consider both the intervention and the actor employing this intervention, though not the transgression type, when making judgments about fairness.

Addressing Hypothesis 3, we did not find a significant effect of transgression type for the majority of the dependent measures. Though our confirmatory analyses did show that children perceived theft as worse than unfairness, for the likelihood of victim forgiveness, evaluations of nonforgiveness, and fairness items, transgression type did not have a significant effect on children's ratings. This pattern of results suggests that transgression severity may not play a role in these judgments, perhaps because the transgressions were all described as intentional or because children were more sensitive to the consequences of the transgression (i.e., the victim having none of the desired resources) rather than the type of transgression.

Addressing Hypothesis 4, we documented a clear preference for compensation across all three actors. We found that children tended to select compensation regardless of which actor was intervening; although their selections of punishment differed based on whether a teacher, peer, or the victim themselves was responding. This pattern of results suggests that children generally view compensation as the normative response to interpersonal transgressions, although their selections are sensitive to authority status and social role.

## 6 General Discussion

The results of Studies 1 and 2 provide evidence that children consider both *who* is intervening and *how* they engage in justice‐oriented interventions when evaluating victim forgiveness. Collectively, these two experiments shed light on how information regarding interventions to interpersonal transgressions is incorporated into children's judgments of how victims will and ought to respond and how victims and transgressors will feel. In addition, our results indicate that children do differentiate in their judgments of the fairness of these various second‐ and third‐party interventions, and that these judgments may be related to perceptions of the likelihood of forgiveness and victims' obligation to forgive.

Participants in both studies also distinguished between justice‐oriented interventions in their expectations of victim forgiveness. In Study 1, victim forgiveness was rated more likely after a third party compensated, punished, or forgave, compared to after they did nothing, and in Study 2, the likelihood of victim forgiveness was rated highest after compensation and lowest after doing nothing (a pattern that remained consistent regardless of who was intervening). These patterns of results suggest that the presence of any intervention increases the perceived likelihood of victim forgiveness, but that victim compensation may be perceived as uniquely effective at promoting forgiveness. Though both punishment and compensation restored an imbalance between the transgressor and victim in our study, children's preference for compensation suggests that they may be more focused on victim welfare, and in particular positive material and emotional outcomes. Although children may prefer, in real‐life contexts, interventions that both punish transgressors and restore property to victims, by studying these interventions independently we can parse apart children's beliefs about the fairness of compensation and punishment as well as their consequences for forgiveness.

In both studies, participants tended to rate victims' decisions to not forgive as bad, although they simultaneously reported that victims were not obligated to forgive. In Study 1, only evaluations of nonforgiveness, and not the victims' obligation to forgive, were sensitive to third‐party intervention, with the victim's choice not to forgive being rated worse after the teacher punished compared to after they did nothing. In Study 2, evaluations of nonforgiveness and obligation to forgive differed in accordance with intervention type, with nonforgiveness being rated worse after compensation or punishment relative to doing nothing, and obligation to forgive being rated higher after punishment and compensation relative to doing nothing.

Although the effect of intervention in Study 2 suggests that evaluations of nonforgiveness and ratings of victims' obligation depend on who intervenes and how, the results from Studies 1 and 2 point to children's views of forgiveness as supererogatory: prosocial but not obligatory. Previous research has found evidence that children understand the distinction between obligatory and supererogatory acts and tend to view failures to complete supererogatory acts as bad (Khan et al. 2023). Our findings align with this previous work on prosocial behavior and show that children are able to apply this framework to forgiveness. Further research is needed to clarify how children balance these ideas about morality and obligation in the context of forgiveness and justice more generally, as these attitudes have implications for how victims are evaluated and treated. In particular, the present study does not allow us to determine how children understand nonforgiveness; it might be that children think of the withholding of forgiveness as akin to holding a grudge against a transgressor or that they think of nonforgiveness as the absence of a response.

Our results provide evidence that children do view compensation as the fairest response to interpersonal transgressions when compared to punishing or pardoning the transgressor or doing nothing. In Study 2, this effect did not differ depending on whether the intervener is a teacher, peer, or victim, or whether the transgression was a theft or fairness violation. Children consistently rated punishment as the second most fair, but their ratings depended on which actor was intervening: children rated punishment as fair from teachers, unfair from victims, and at chance levels for peers. This finding aligns with previous work documenting children's differential evaluation of second‐ and third‐party punishment (Strauß and Bondü 2022) and also sheds light on what factors may contribute to children's decisions to opt for punishment as third parties. Moreover, children's ratings of punishment as fairer than pardoning or doing nothing support theorizing by Bilz (2016) and results from Bregant et al. (2016): children seem to prefer punishment over inaction or the condonation of antisocial behavior. In addition, we found that in Study 1, the majority of participants ranked compensation as the fairest, and in Study 2, participants most frequently reported that actors should compensate victims. These results further bolster our claims that children prefer victim‐oriented over transgressor‐oriented interventions and also provide evidence that this preference is linked to fairness judgments.

## 7 Limitations

Although the present studies offer new insights into children's beliefs about transgressions, interventions, and forgiveness, there exist several limitations that must be acknowledged in discussing the results. First, our studies included samples of participants that were recruited only in the United States and who were the majority White and from liberal, high SES families. Future research should explore cultural variation in children's beliefs about interventions and forgiveness, as well as how parental factors (e.g., religiosity, political affiliation) may predict children's beliefs. Previous research suggests that, although children across cultures exhibit similar levels of forgiveness behavior (Amir et al. 2024), cultural context may shape how children (Vera Cruz et al. 2024) and adults (Joo et al. 2019; Ho and Worthington Jr 2020) understand forgiveness.

Second, due to the format of the studies and ages of the participants, we had to restrict the range of intervention conditions and dependent variables. It may be possible that children hold different beliefs about other interventions, such as corporal punishment, consolation of victims, or mediation. Moreover, we neglected to include other dependent variables that may be of interest, including the likelihood of reconciliation, recidivism, or intervener motives. Future studies can explore these additional questions in similar paradigms to further expand our understanding.

Third, the present studies only measured children's evaluations and expectations of others' behavior, rather than measuring children's own behavior when they are victims of transgressions. It may be that, although children expect victims to be more likely to forgive after some interventions, they themselves would not alter their forgiveness behavior. Examining these questions with behavioral tasks will provide critical insights into how interventions actually affect victims' behavior, which may in turn help shape advice for parents, teachers, and other adults as they mediate conflicts between children.

Despite these limitations, we believe that the current studies contribute valuable new information about children's views on transgressions, interventions, justice, and forgiveness. Our results suggest that children hold strong beliefs regarding the fair responses to transgressions from both second and third parties and that these beliefs map onto their expectations of victims' forgiveness. We found evidence that children view forgiveness as supererogatory—rating the decision to refrain from forgiveness as bad but still perceiving victims as not obligated to forgive regardless of intervention. These findings suggest that interventions and justice‐related pursuits may play an important role in children's decisions to engage in forgiveness or other post‐transgression behaviors and also point to the importance of further studying contextual factors that contribute to children's decisions to forgive and evaluations of forgiveness.

## 8 Conclusion

The results of the present studies generate valuable, novel insights into how children evaluate both interventions and victims' behavior following these interventions. Previous research has largely focused on children's forgiveness‐related behavior after apologies or reparations from transgressors (e.g., Oostenbroek and Vaish 2019a; Amir et al. 2021). In the present studies, we sought to instead explore how interventions from third parties or victims influenced participants' evaluations and expectations of forgiveness‐related outcomes. Our findings show that children take these factors into account when rating victims' likelihood of forgiveness, obligations to forgive, and nonforgiveness, and point to the need to further study justice and forgiveness in ecologically valid contexts. These findings have important implications for educators, parents, and others who work with children—if we want to cultivate positive, forgiving relationships between children and avoid the potential consequences of encouraging forgiveness when not warranted, we ought to respond to their conflicts in ways that reflect their perceptions of justice. Doing so can help children to feel empowered and serve to repair relationships in ways that are fair and satisfying for all involved parties.

## Conflicts of Interest

The authors declare no conflicts of interest.

## Supporting information


**Data S1:** cdev70030‐sup‐0001‐supinfo.docx.

## Data Availability

The data and code necessary to reproduce the analyses presented here are publicly accessible, as are the materials necessary to attempt to replicate the findings. Analyses were also preregistered. Data, code, materials, and the preregistration for this research are available at the following URL: https://osf.io/xr4be/files/osfstorage?view_only=4685b21367e848708d09b10c6a5e7832.
