# Supplementary material for: Children's Evaluations and Expectations of Forgiveness Following Second‐ and Third‐Party Interventions
Source: Child Dev. 2025 Aug 21;96(6):2146–61. doi: 10.1111/cdev.70030 (PMC12598450; doi:10.1111/cdev.70030)
Supplement: Supplementary file 1 — Data S1: cdev70030‐sup‐0001‐supinfo.docx. [file CDEV-96-2146-s001.docx]

**Table of Contents**

Age Effects for the Likelihood of Forgiveness 2

Age Effects for Evaluations of Non-Forgiveness 2

Age Effects for Obligation to Forgive 2

Age Effects for Victim Emotions 2

Age Effects for Offender Emotions 3

Age Effects for Fairness 3

Relationship between fairness and forgiveness-related evaluations (Study 1) 4

Confirmatory analyses for transgression severity (Study 2). 5

Relationship between fairness and forgiveness-related evaluations (Study 2) 6

Figure S1. Fairness rankings of interventions in Study 1 7

Figure S2. Age effects for Likelihood of Forgiveness, Obligation to Forgive, Evaluations of 8

Non-Forgiveness, and Fairness in Study 2.

Figure S3. Likelihood of Victim Forgiveness by Transgression Type. 9

Figure S4. Obligation to Forgive by Transgression Type. 10

Figure S5. Evaluations of Non-Forgiveness by Transgression Type. 11

Figure S6. Fairness by Transgression Type. 12

Table S1. Model output for Likelihood of Forgiveness in Study 1. 13

Table S2. Model output for Evaluations of Non-Forgiveness in Study 1. 14

Table S3. Model output for Obligation to Forgive in Study 1. 15

Table S4. Model output for Victim Emotions in Study 1. 16

Table S5. Model output for Offender Emotions in Study 1. 17

Table S6. Model output for Fairness in Study 1. 18

Table S7. Model output for Likelihood of Forgiveness in Study 2. 19

Table S8. Model output for Morality of Non-Forgiveness in Study 2. 20

Table S9. Model output for Obligation to Forgive in Study 2. 21

Table S10. Model output for Fairness in Study 2. 22

OSF Link 23

**Study 1 Age Effects**

**Age Effects for Likelihood of Forgiveness**

Participants’ ratings also differed with age; our second model, which included the interaction between Age and Intervention, was a better fit than our model including just the main effect of Intervention (*𝛸^2^*(3) = 20.64, *p*  < .001). Children increasingly differentiated between the likelihood of forgiveness after compensating (*𝛽* = 0.04, *p* < .001), punishing (*𝛽* = 0.05, *p* < .001), and pardoning (*𝛽* = 0.03, *p* = .006) compared to doing nothing with age.

**Age Effects for Evaluations of Non-Forgiveness**

Participants’ ratings did not systematically vary with age; our second model including the interaction between Age and Intervention was not a better fit than our model including just the main effect of Intervention (*X^2^*(3) = 1.83, *p*  = .609). As children got older, they continued to report that non-forgiveness was bad, and this trend did not vary based on how the teacher intervened.

**Age Effects for Obligation to Forgive**

Participants’ patterns of responses also differed with age; our second model, including the interaction between Age and Intervention, was a better fit than our model including just the main effect of Intervention (*X^2^*(3) = 11.52, *p*  = .009). With age, children increasingly differentiated between the victim’s obligation to forgive after compensating (*𝛽* = 0.02, *p* = .022) and punishing (*𝛽* = 0.03, *p* = .001) compared to doing nothing.

**Age Effects for Victim Emotions**

Participants’ ratings of victim emotions also varied with age; our second model, which included the interaction between Age and Intervention, was a better fit than the model including only the main effect of Intervention (*X^2^*(3) = 11.75, *p*  = .008). In particular, children increasingly differentiated between victim emotions after the teacher compensated (*𝛽* = 0.02, *p* = .044) and punished (*𝛽* = 0.03, *p* = .004) compared to after the teacher did nothing with age.

**Age Effects for Offender Emotions**

Participants’ ratings of offender emotions also varied with age; our second model, including the interaction between Age and Intervention, was a better fit than the main effects model (*X^2^*(3) = 8.92, *p*  = .030). Children increasingly differentiated between offender emotions after the teacher punished compared to after the teacher did nothing with age (*𝛽* = -0.03, *p* = .003), rating offenders as feeling sadder after punishment with age.

**Age Effects for Fairness**

Our second model, which included the interaction between Age and Intervention, was a better fit than our main effects model (*X^2^*(3) = 13.18, *p*  = .004), suggesting that children’s evaluations of the fairness of the interventions varied with age. We found a significant interaction between Age and punishment (*𝛽* = 0.03, *p* = .008), indicating that, relative to doing nothing, children rated punishment as increasingly fair with age.

**Exploratory Analyses with Children’s Fairness Responses**

In addition to our pre-registered analyses, we conducted two exploratory analyses: first, to examine children’s rankings of the fairness of the four interventions, and second, to investigate the relationship between judgments of the fairness of the intervention and the other three dependent variables. By doing so, we were able to test if, in alignment with the adult literature (e.g., Wenzel & Okimoto, 2013), children’s evaluations of how fair the intervention was predicted their expectations and evaluations of forgiveness. To look at children’s fairness rankings, we performed a Kruskal-Wallis rank sum test based on rankings of the four interventions. Although children most frequently rated compensation and pardoning to be most fair and doing nothing and punishment as least fair, the results of the test were not significant (*X*^2^(3) = 6.99, *p* = .072), suggesting that children did not significantly distinguish between the four interventions in their rankings. This may be due to challenges with the questions, including children’s memory of each story, so we will refrain from making much of these results.

In our second exploratory analysis, we ran five linear mixed effects models to test if children’s fairness ratings predicted their ratings for each of the other dependent measures (Likelihood of Forgiveness, Obligation to Forgive, and Morality of Non-Forgiveness). We ran linear mixed effects models with Fairness, Age, and Gender as fixed effects and participant ID as a random effect. We found that, in fact, fairness ratings did predict children’s ratings for all other dependent measures (*p* < .05). In particular, children’s fairness ratings positively predicted their ratings of the likelihood of victim forgiveness (*𝛽* = 0.20, *p* < .001), obligation to forgive (*𝛽* = 0.09, *p* = .016), and victim emotions (*𝛽* = 0.60, *p* < .001), and negatively predicted ratings of the morality of non-forgiveness (*𝛽* = -0.07, *p* = .027) and offender emotions (*𝛽* = -0.25, *p* < .001).

**Study 2**

**Confirmatory Analyses for Transgression Severity**

After looking at results for each of the main dependent variables, we also conducted confirmatory analyses to ensure that participants differentiated between fairness violations and theft. At the end of the experimental session, participants once again saw the stimuli for one of the vignettes and were asked to rate how good or bad the transgression (theft or fairness violation) was (-3 = very bad, +3 = very good), and then were asked how good or bad it would have been if the transgressor had committed the other transgression type (-3 = very bad, +3 = very good). After rating the morality of each transgression separately, participants were then asked which was worse (theft or not sharing), as well as how much worse (a tiny bit, a little bit, a lot). Responses were coded from -3 (not sharing much worse than theft) to +3 (theft much worse than not sharing). We first conducted an unpaired two-sample t-test comparing participants’ ratings of the morality of the theft and fairness violations. We failed to find a significant effect of transgression type (*t* = 0.60, *p*  = .547), with theft transgressions (*M* = -2.65) being rated only marginally worse than fairness transgressions (*M* = -2.60) on the continuous measure. We next conducted a one-sample t-test comparing responses for which transgression was worse to chance and found that participants were more likely to say that theft was worse than not sharing (*t* = 3.73, *p* < .001, *M* = 0.68). This result provides some evidence that participants did, in fact, view theft to be worse than not sharing when forced to choose, although this difference in severity did not seem to affect results for the majority of the dependent variables.

**Relationship between Fairness and Forgiveness-Related Evaluations**

In addition to our pre-registered analyses, as in Study 1, we conducted exploratory analyses to investigate the relationship between judgments of the fairness of the intervention and the other three dependent variables. By doing so, we were able to test if, in alignment with the adult literature (e.g., Wenzel & Okimoto, 2013), children’s evaluations of how fair the intervention was predicted their expectations and evaluations of forgiveness. We ran three linear mixed effects models with Fairness predicting each of the other dependent measures (Likelihood of Forgiveness, Obligation to Forgive, and Morality of Non-Forgiveness) and included Age and Gender as fixed effects and Participant ID as a random effect.

We found that fairness judgments only predicting ratings of the likelihood of forgiveness (*𝛽* = 0.15, *p* < .001); neither the relationship between fairness and morality of non-forgiveness (*𝛽* = 0.00, *p* = .080) nor between fairness and obligation to forgive (*𝛽* = 0.00, *p*  = .695) was significant. This result indicates that, like adults (Wenzel & Okimoto, 2014), children make predictions about when forgiveness will occur based on the extent to which interventions promote justice in the aftermath of a transgression. These post-hoc analyses allowed us to explore the extent to which children’s views of the justice (fairness) of an intervention predicted their fairness-related ratings. Our finding that fairness ratings predicted ratings of the likelihood of forgiveness aligns with previous adult research indicating that activating justice (Karremans & van Lange, 2005) or enacting justice (Wenzel & Okimoto, 2013) can be effective in promoting forgiveness.

**Supplementary Figures**

**Figure S1.** Fairness rankings of Interventions in Study 1.

**
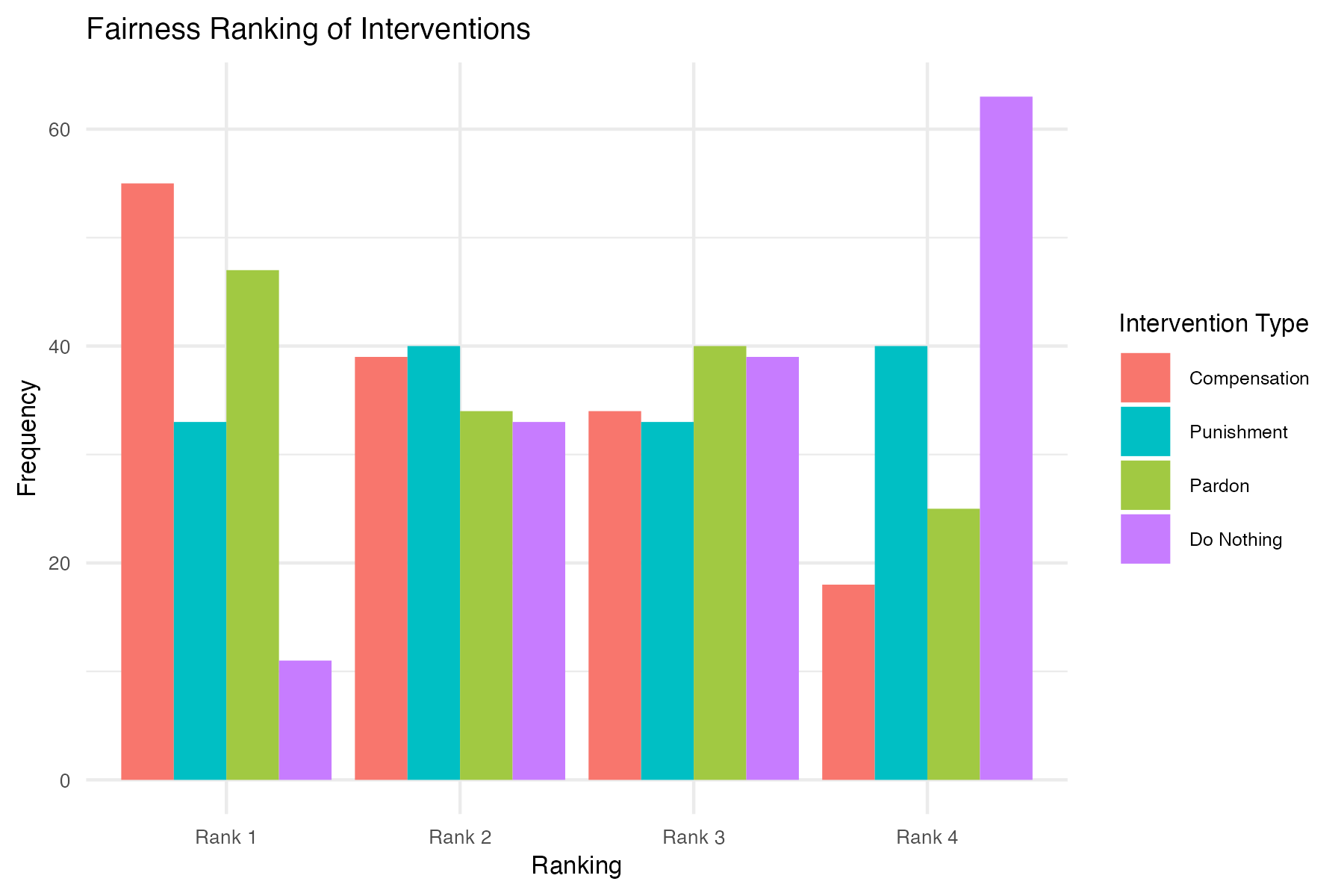
**

***Note.*** Participant rankings of the four interventions (Compensation, Punishment, Pardoning, and Doing Nothing) in terms of fairness. Y-axis represents frequency of selection for each rank position and X-axis represents rank position (Rank 1 - 4).

**Figure S2.** Age effects for Likelihood of Forgiveness, Obligation to Forgive, Evaluations of Non-Forgiveness, and Fairness in Study 2.


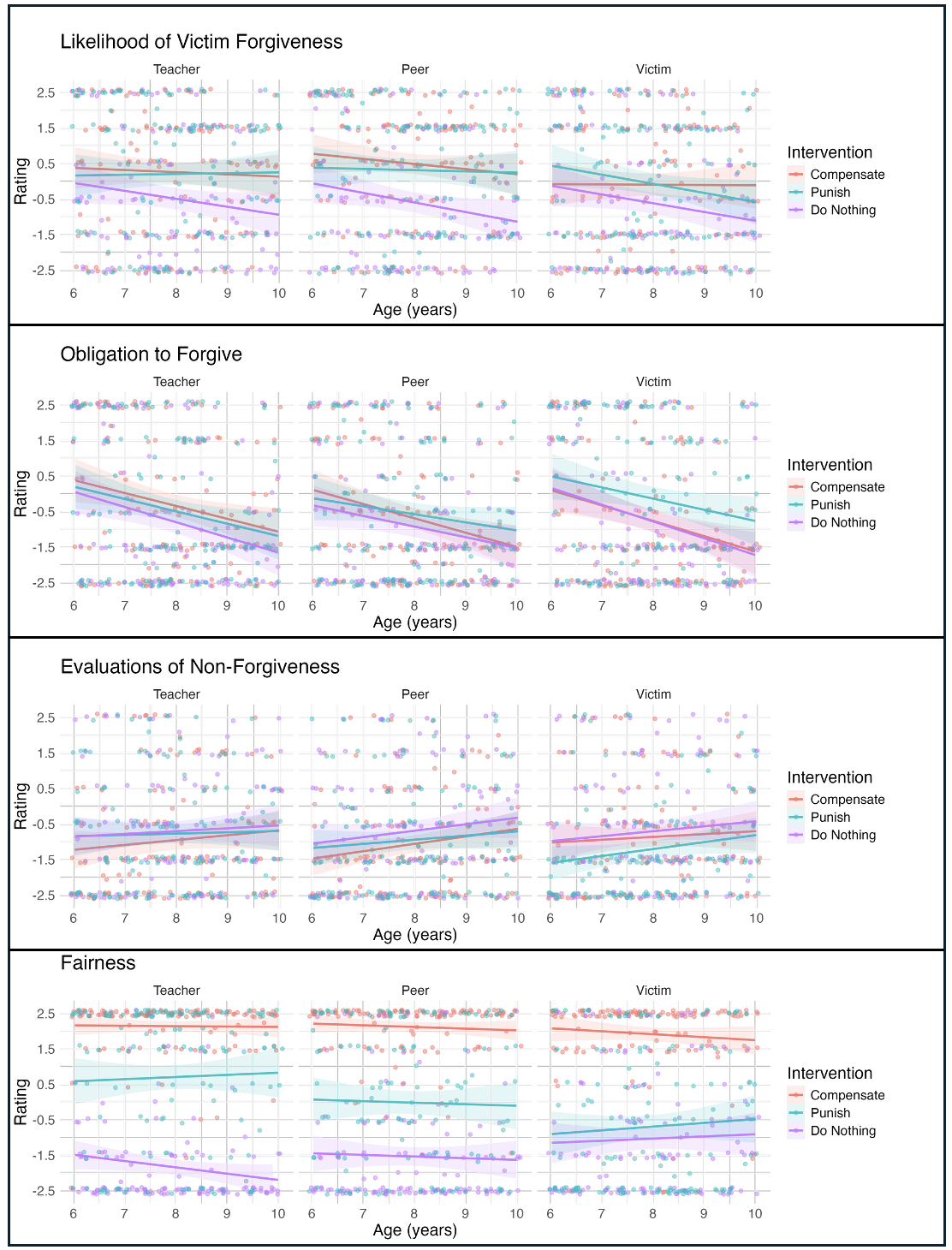


***Note***. Participant ratings of the likelihood of forgiveness, obligation to forgive, and evaluations of non-forgiveness by Intervention (Compensation, Punishment, Doing Nothing), Actor (Teacher, Peer, Victim), and Age (in years). The y-axis shows participant ratings, ranging from -2.5 (very sure no/very bad) to +2.5 (very sure yes/very good) and the x-axis shows age in years. Ribbons represent 95% confidence intervals.

**Figure S3.** *Likelihood of Victim Forgiveness by Transgression Type.*


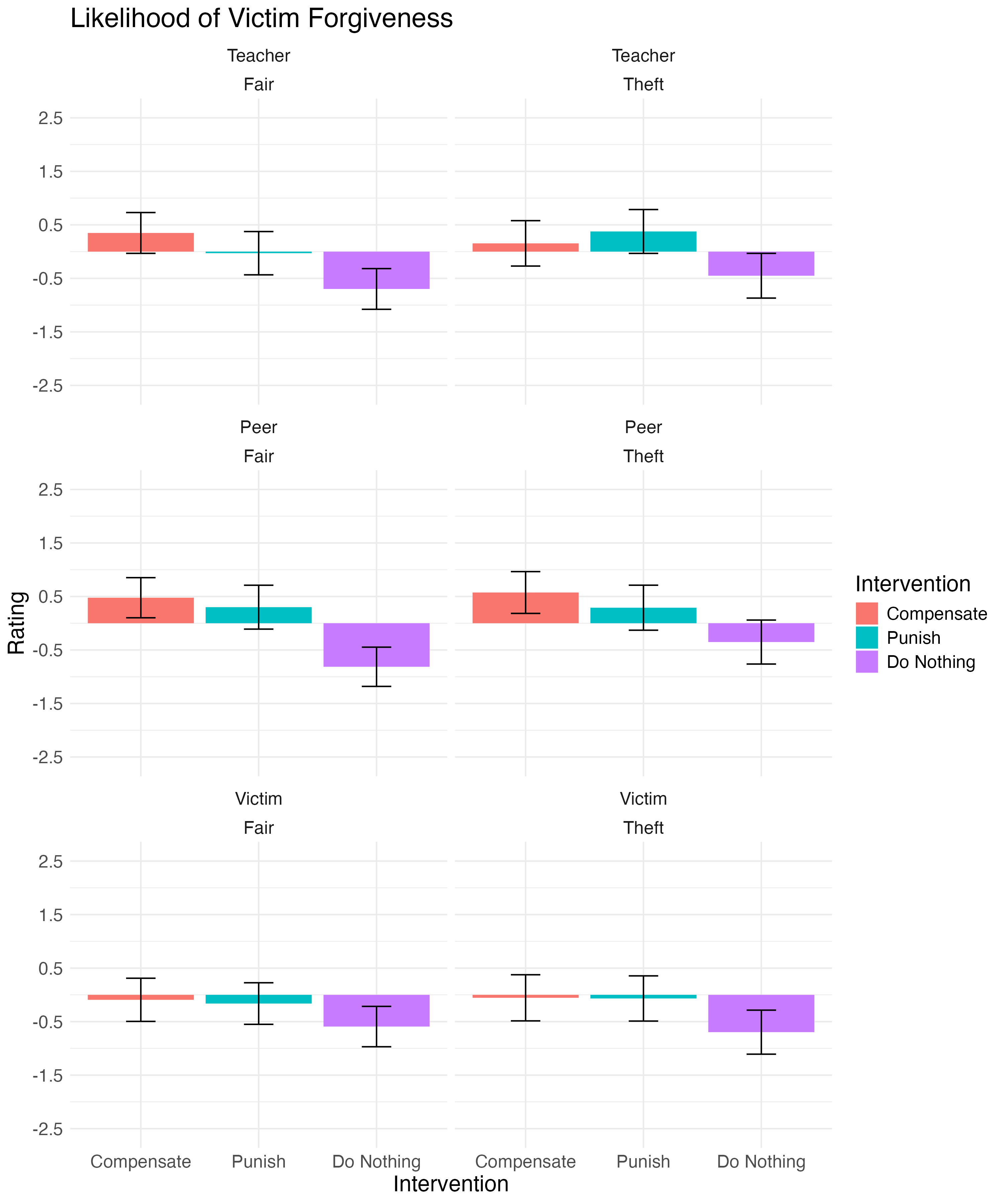


***Note***. Participant ratings for Likelihood of Forgiveness by Intervention (Compensation, Punishment, Doing Nothing), Actor (Teacher, Peer, Victim), and Transgression Type (Unfairness, Theft). The y-axis shows participant ratings, ranging from -2.5 (very sure no/very bad) to +2.5 (very sure yes/very good) and the x-axis shows intervention. Error bars represent 95% confidence intervals.

**Figure S4.** *Obligation to Forgive by Transgression Type.*


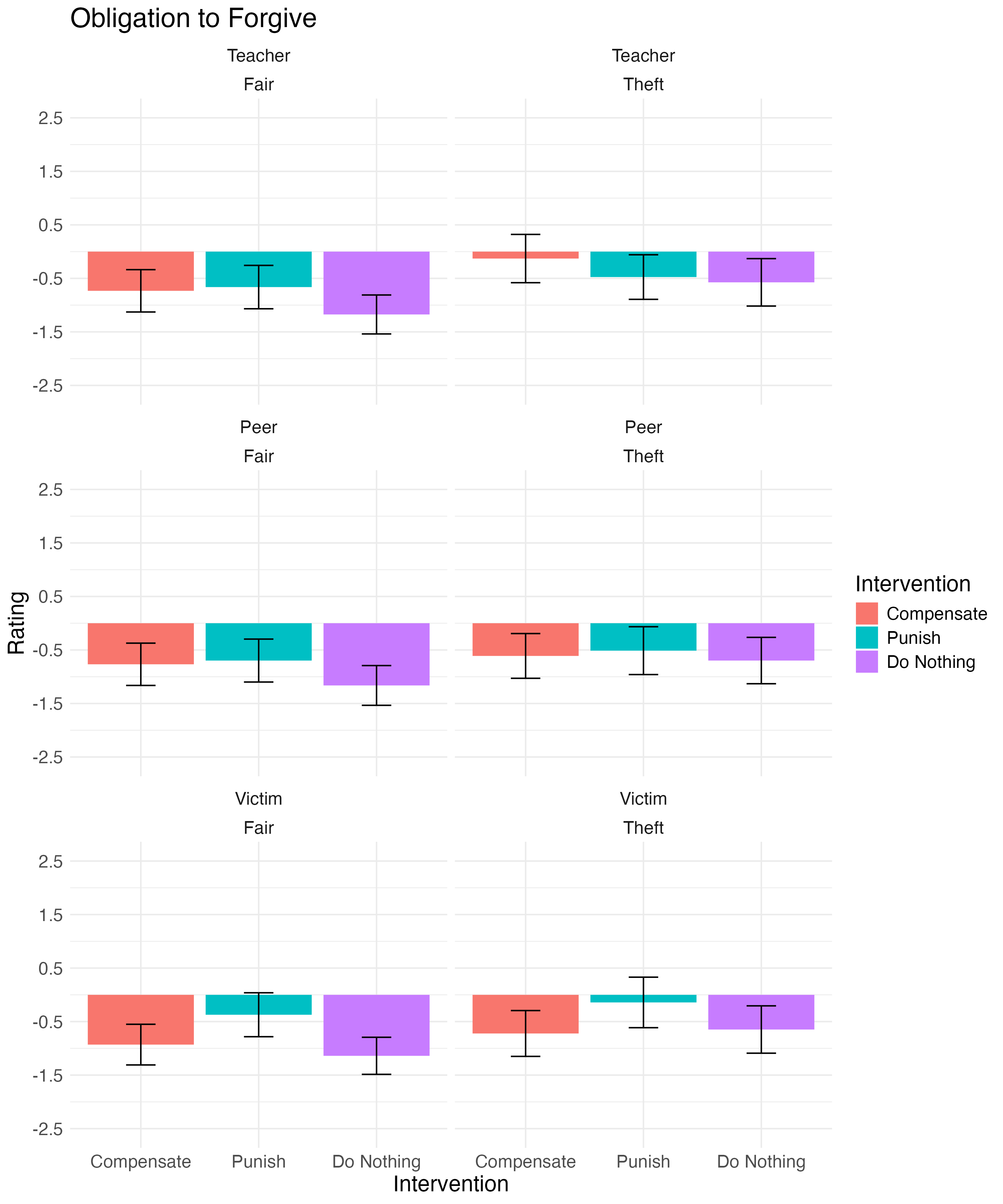


***Note***. Participant ratings for Obligation to Forgive by Intervention (Compensation, Punishment, Doing Nothing), Actor (Teacher, Peer, Victim), and Transgression Type (Unfairness, Theft). The y-axis shows participant ratings, ranging from -2.5 (very sure no/very bad) to +2.5 (very sure yes/very good) and the x-axis shows intervention. Error bars represent 95% confidence intervals.

**Figure S5.** *Evaluations of Non-Forgiveness by Transgression Type.*


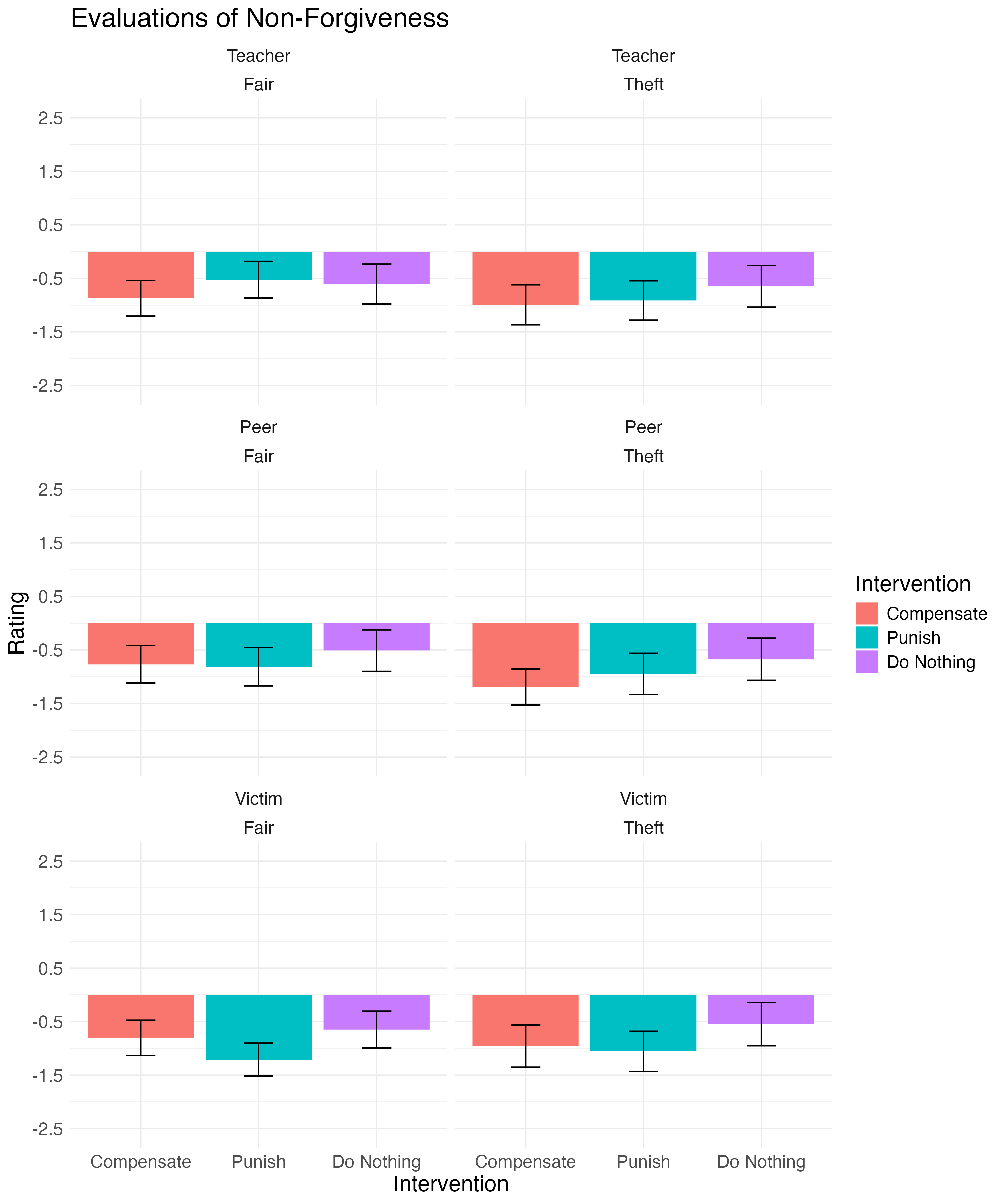


***Note***. Participant ratings for Evaluations of Non-Forgiveness by Intervention (Compensation, Punishment, Doing Nothing), Actor (Teacher, Peer, Victim), and Transgression Type (Unfairness, Theft). The y-axis shows participant ratings, ranging from -2.5 (very sure no/very bad) to +2.5 (very sure yes/very good) and the x-axis shows intervention. Error bars represent 95% confidence intervals.

**Figure S6.** *Fairness by Transgression Type.*


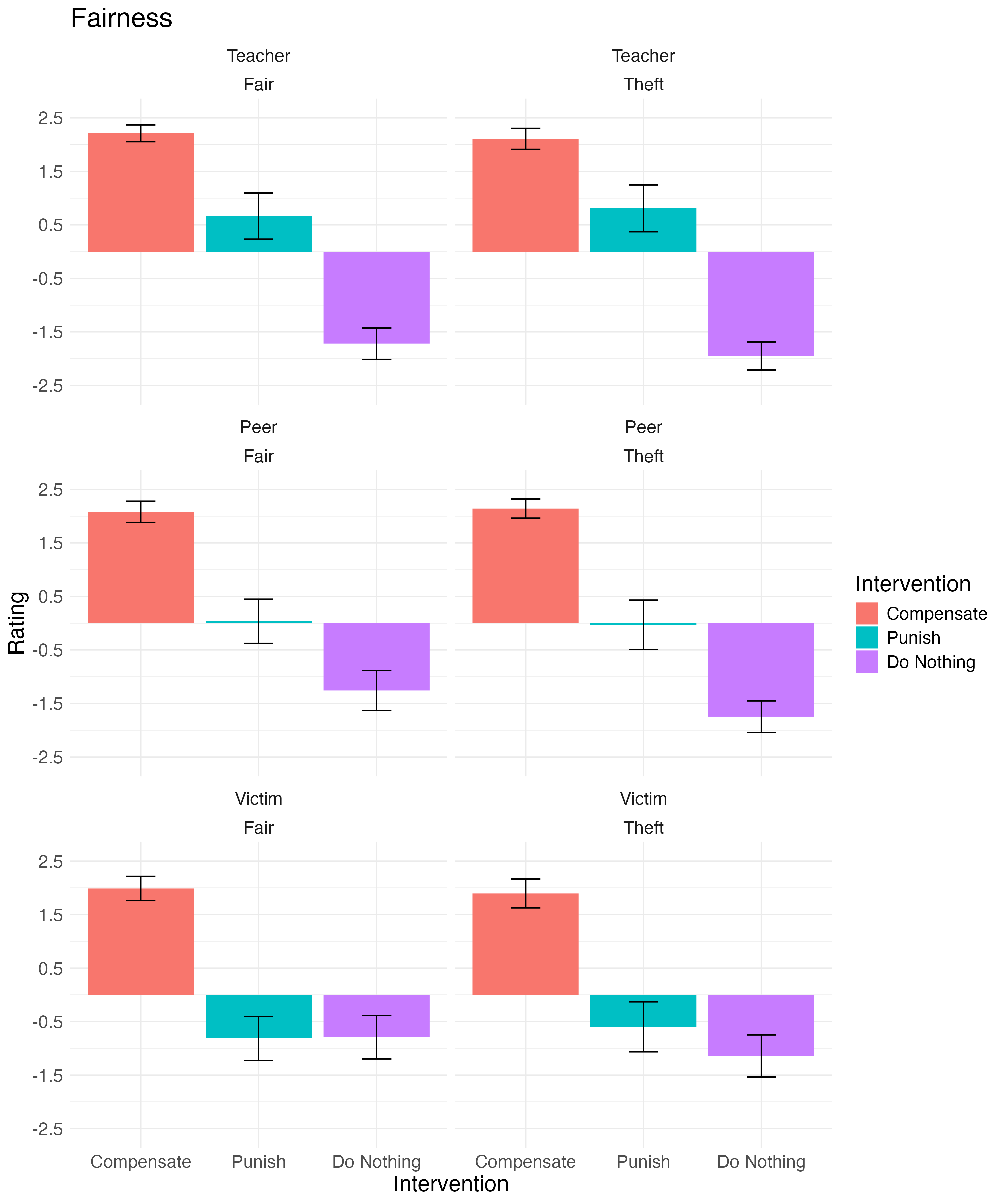


***Note***. Participant ratings for Fairness by Intervention (Compensation, Punishment, Doing Nothing), Actor (Teacher, Peer, Victim), and Transgression Type (Unfairness, Theft). The y-axis shows participant ratings, ranging from -2.5 (very sure no/very bad) to +2.5 (very sure yes/very good) and the x-axis shows intervention. Error bars represent 95% confidence intervals.

**Supplementary Tables**

**Table S1.** Model output for Likelihood of Forgiveness in Study 1**.**


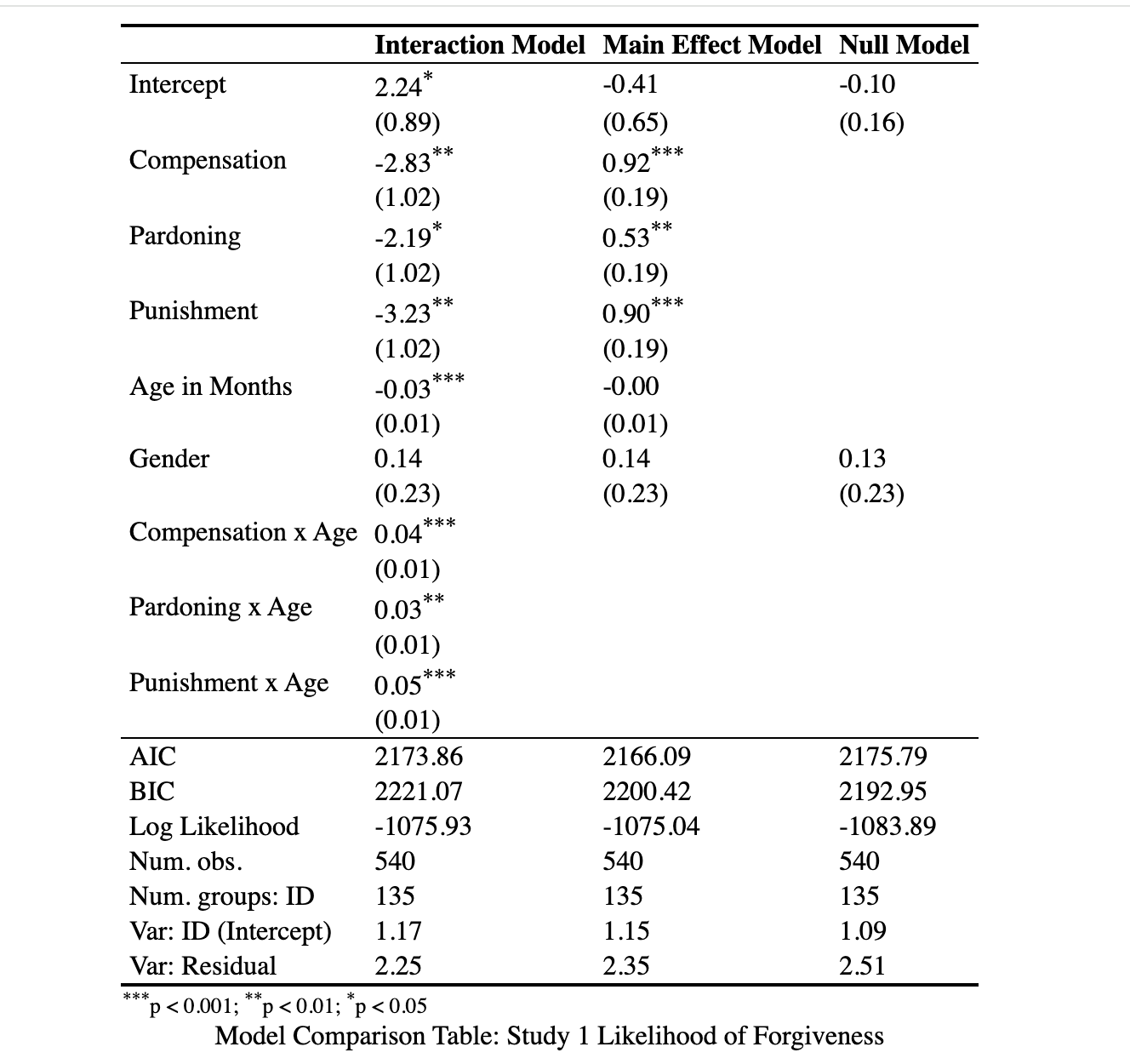


**Table S2.** Model output for Evaluations of Non-Forgiveness in Study 1.


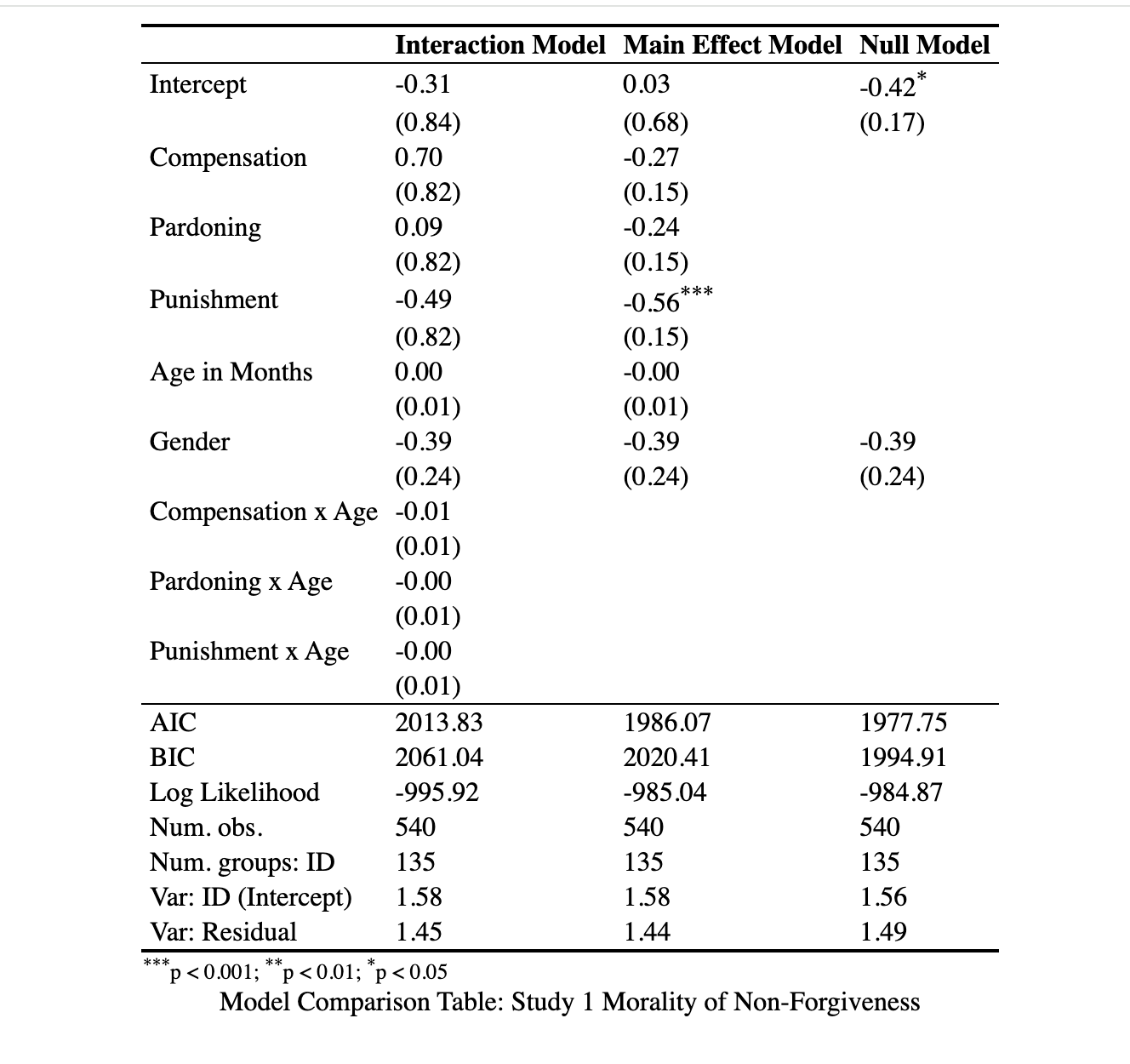


**Table S3.** Model output for Obligation to Forgive in Study 1.


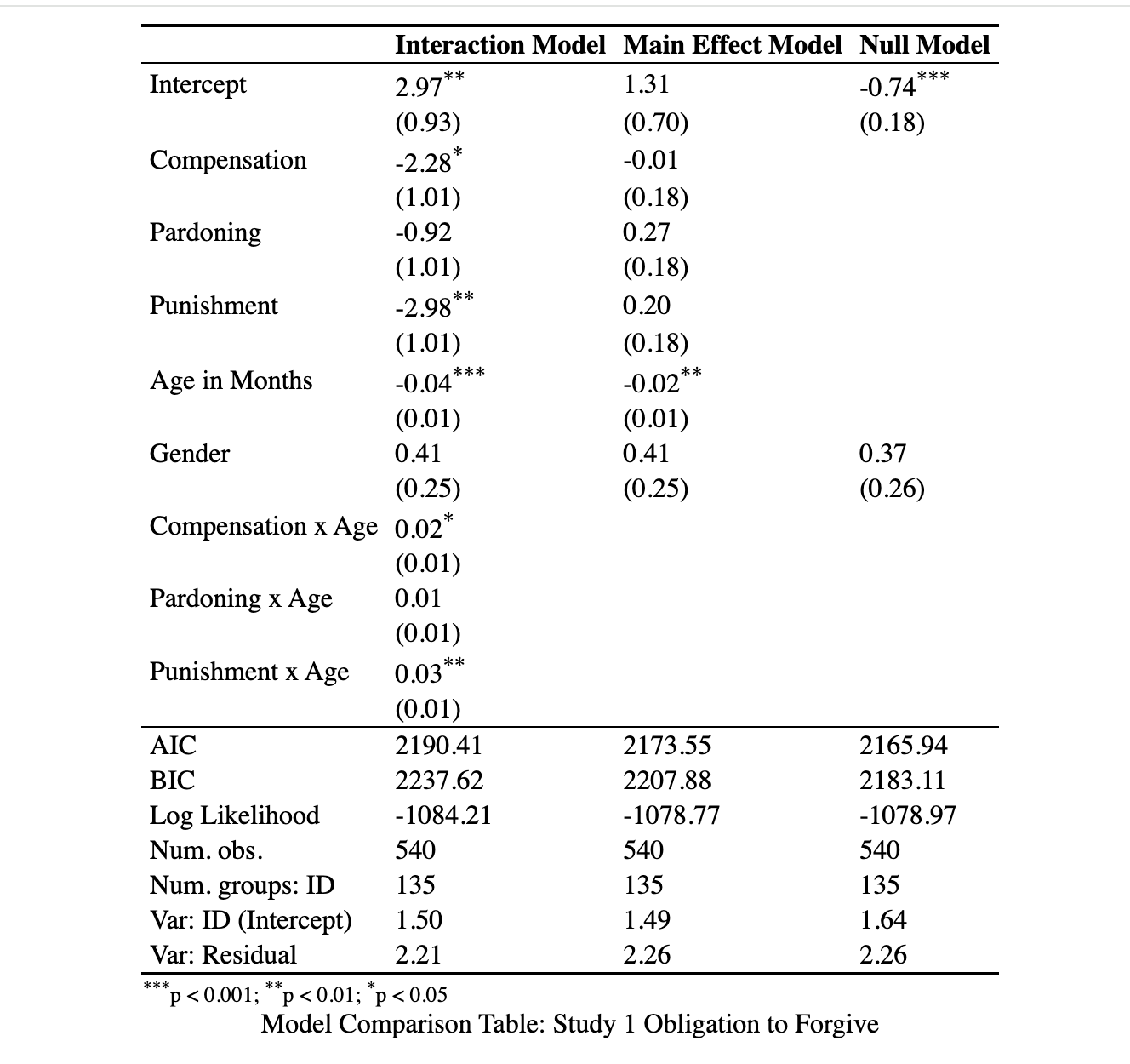


**Table S4.** Model output for Victim Emotions in Study 1.


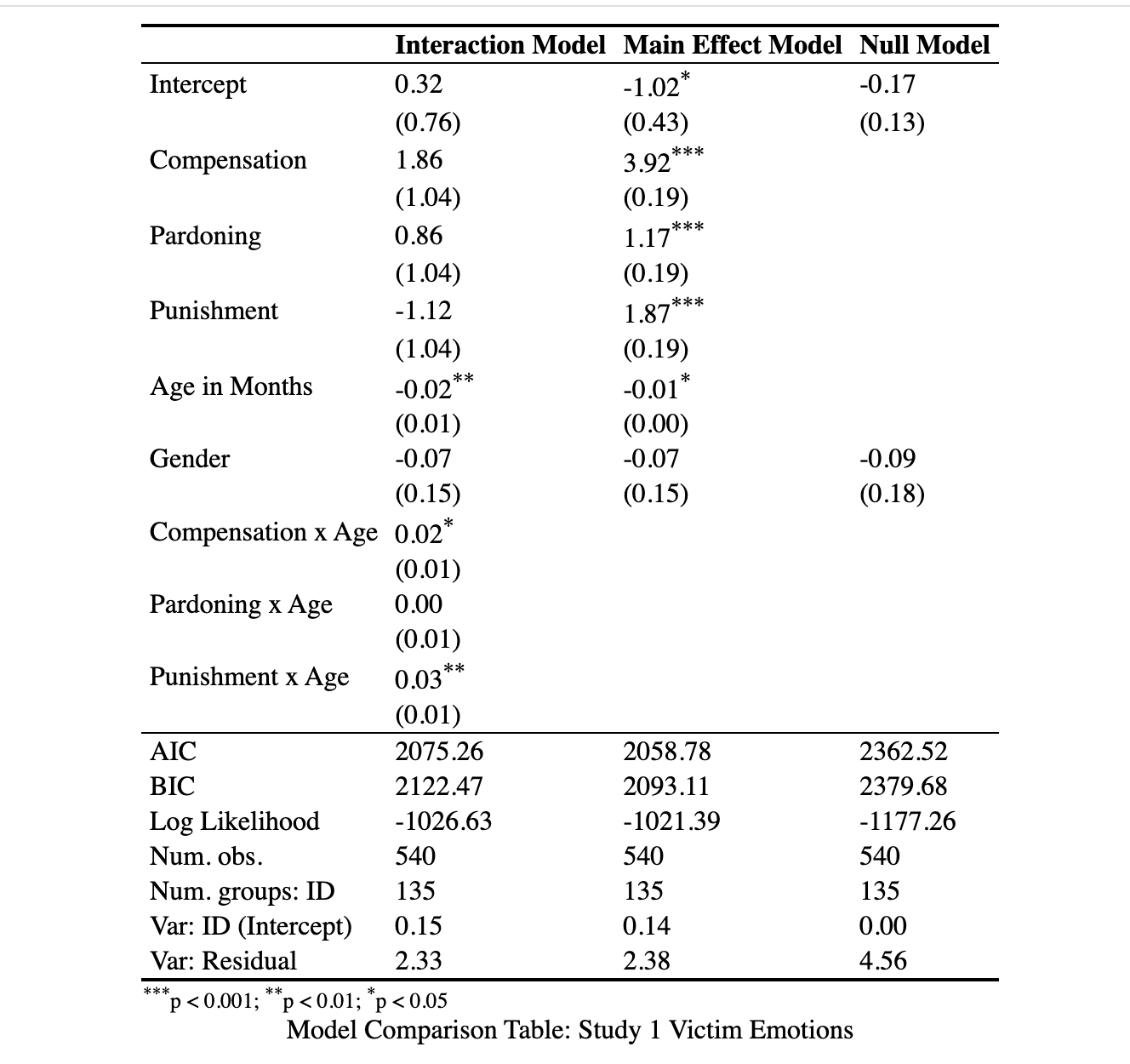


**Table S5.** Model output for Offender Emotions in Study 1.


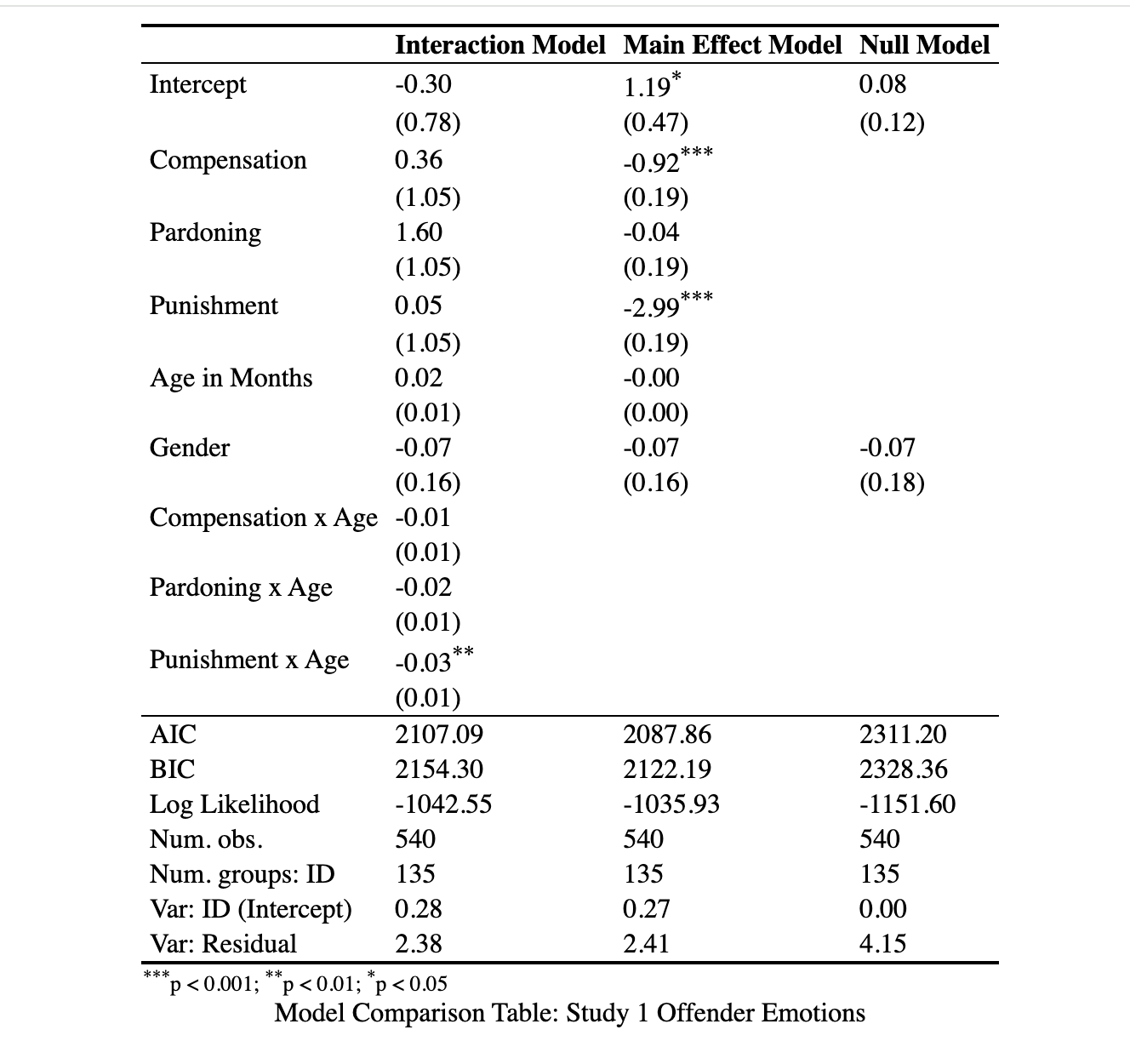


**Table S6.** Model output for Fairness in Study 1.


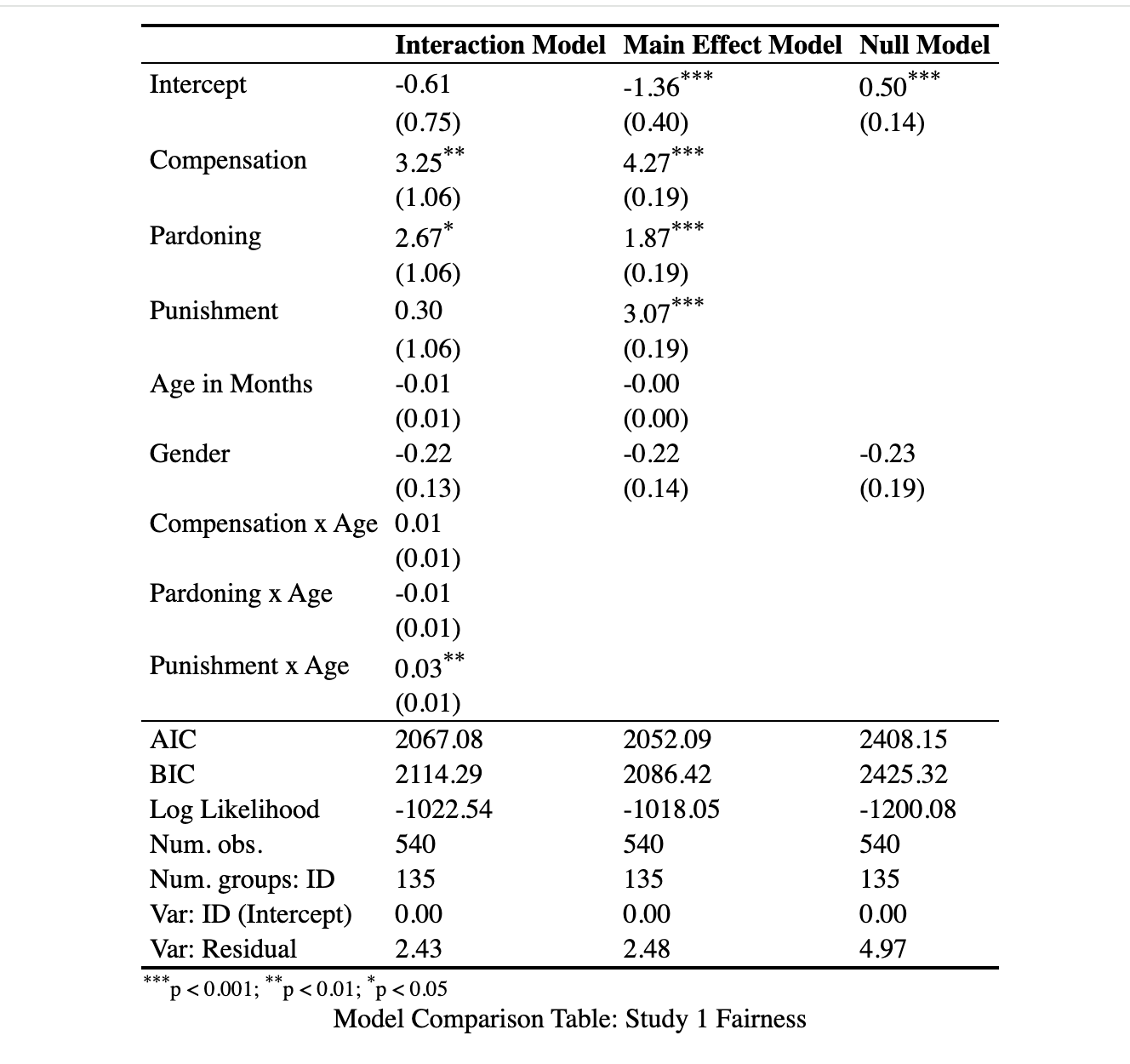


**Table S7.** Model output for Likelihood of Forgiveness in Study 2.


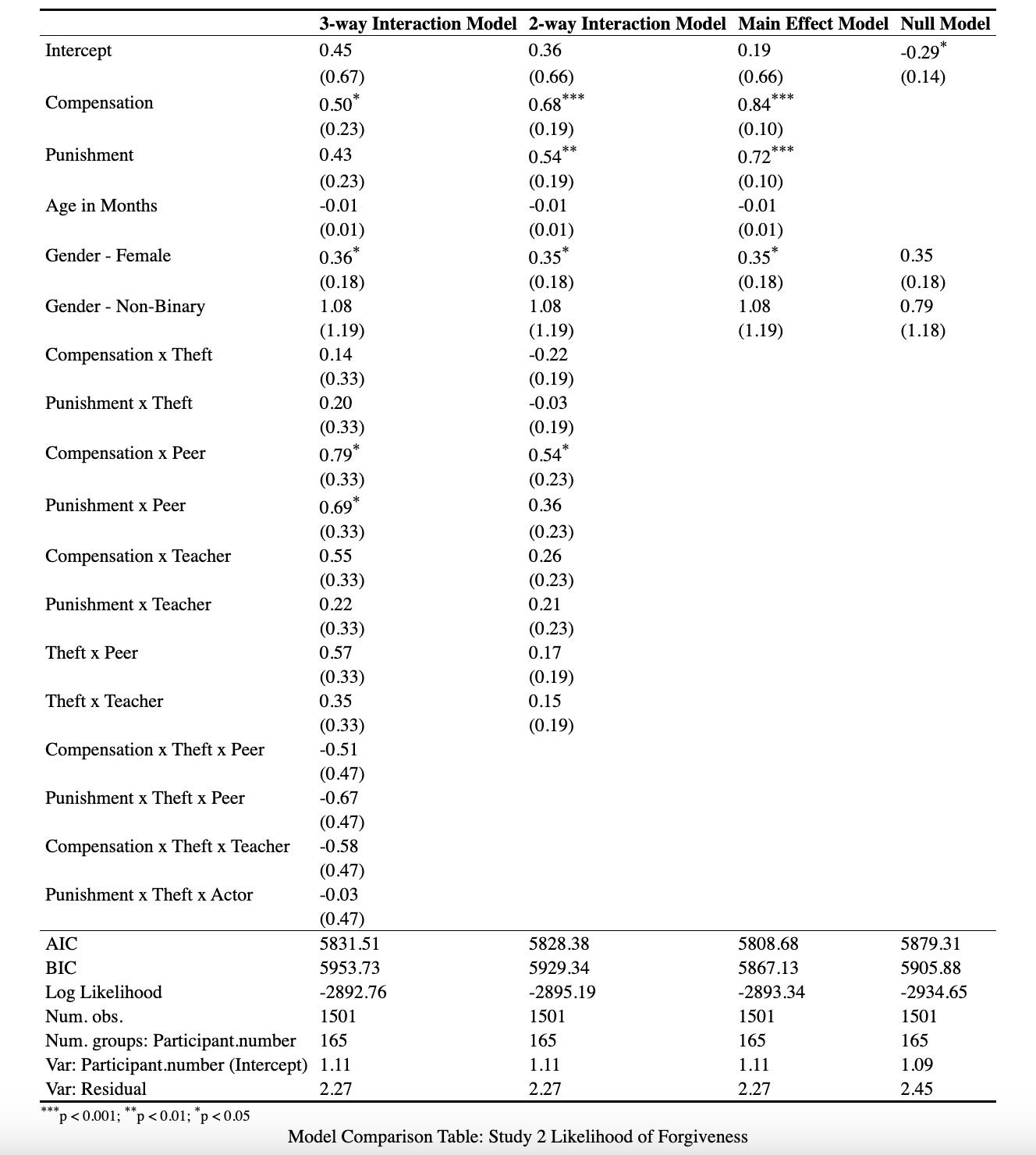


**Table S8.** Model output for Morality of Non-Forgiveness in Study 2.


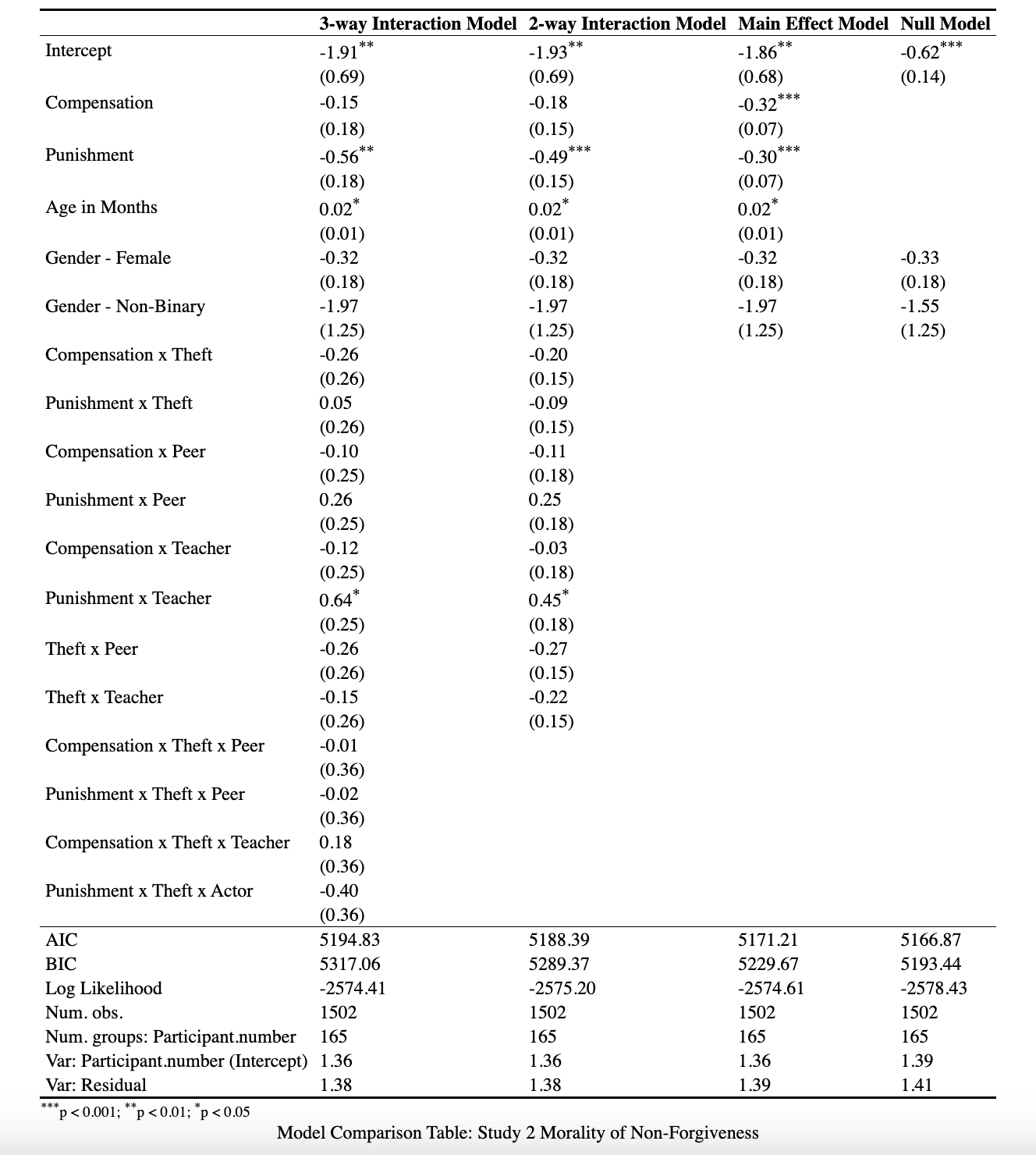


**Table S9.** Model output for Obligation to Forgive in Study 2.


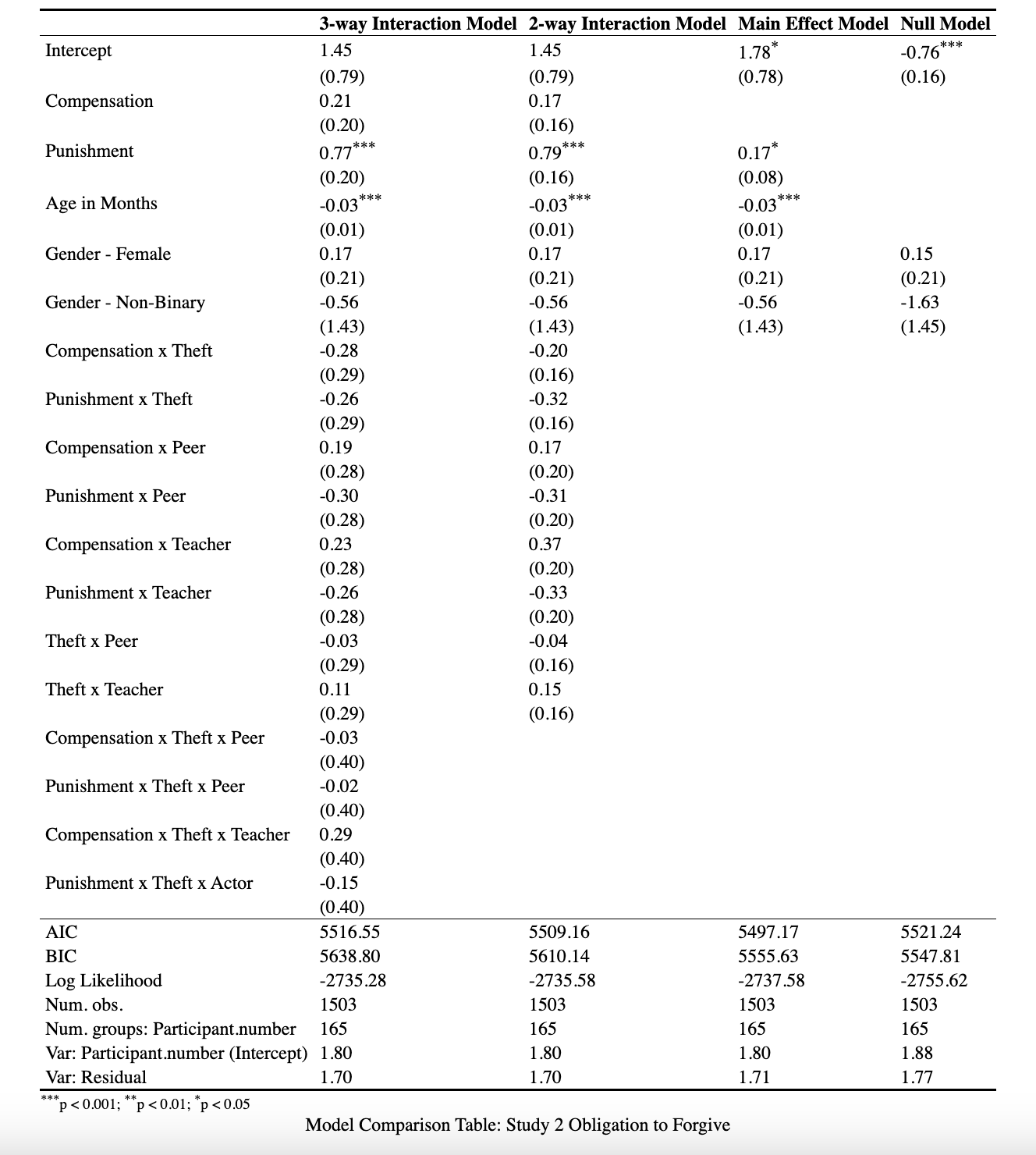


**Table S10.** Model output for Fairness in Study 2.


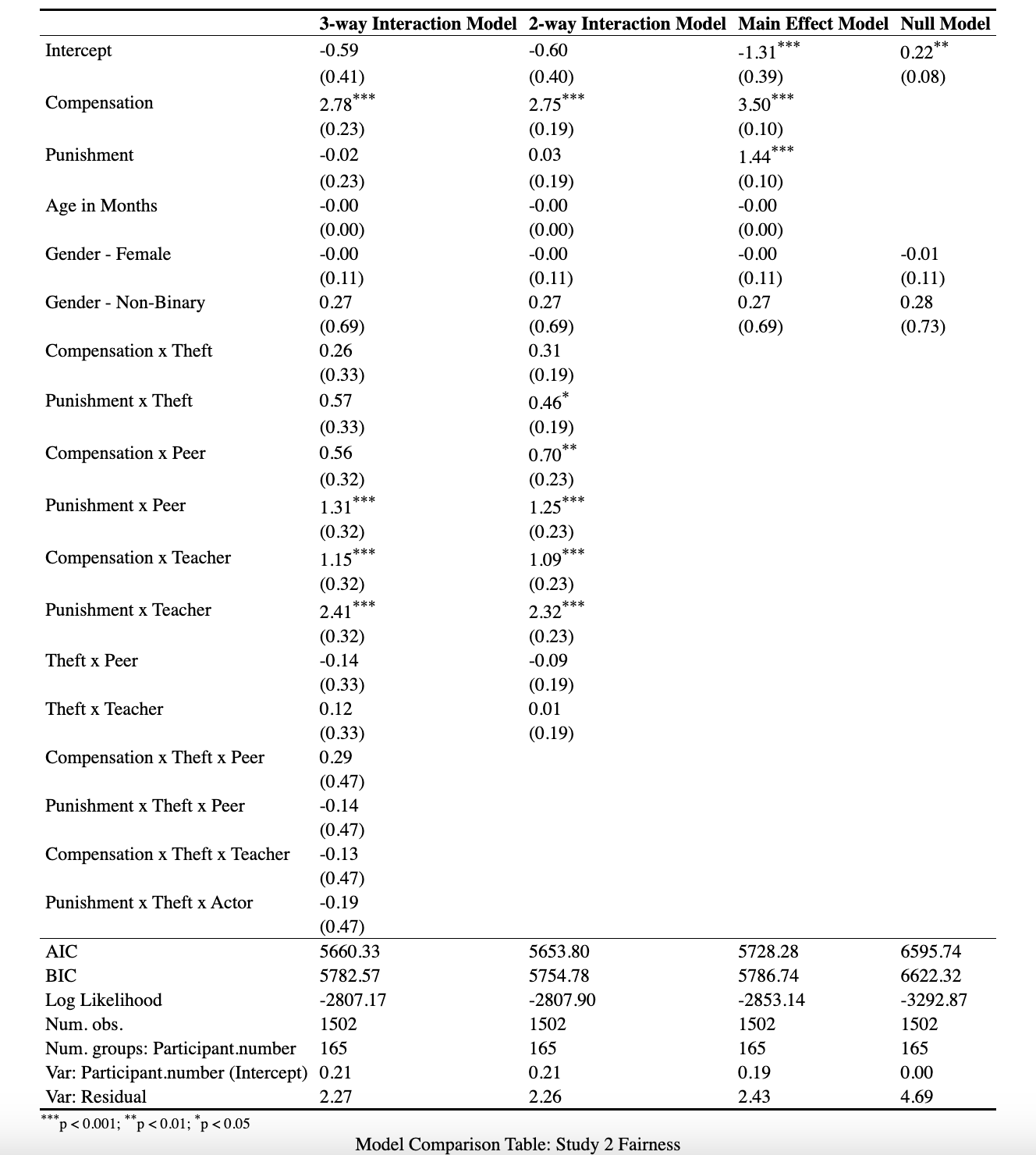


**OSF Link:**

<https://osf.io/xr4be/?view_only=4685b21367e848708d09b10c6a5e7832>
